# Supplementary material for: Opposite Modulation of RAC1 by Mutations in TRIO Is Associated with Distinct, Domain-Specific Neurodevelopmental Disorders
Source: Am J Hum Genet. 2020 Feb 27;106(3):338–55. doi: 10.1016/j.ajhg.2020.01.018 (PMC7058823; doi:10.1016/j.ajhg.2020.01.018)
Supplement: Document S1. Figures S1–S4 and Tables S2 and S3 [file mmc1.pdf]

The American Journal of Human Genetics, Volume *106*

Supplemental Data

Opposite Modulation of RAC1 by Mutations in *TRIO* Is Associated with Distinct, Domain-Specific Neurodevelopmental Disorders

Sónia Barbosa, Stephanie Greville-Heygate, Maxime Bonnet, Annie Godwin, Christine Fagotto-Kaufmann, Andrey V. Kajava, Damien Laouteouet, Rebecca Mawby, Htoo Aung Wai, Alexander J.M. Dingemans, Jayne Hehir-Kwa, Marjorlaine Willems, Yline Capri, Sarju G. Mehta, Helen Cox, David Goudie, Fleur Vansenne, Peter Turnpenny, Marie Vincent, Benjamin Cogné, Gaëtan Lesca, Jozef Hertecant, Diana Rodriguez, Boris Keren, Lydie Burglen, Marion Gérard, Audrey Putoux, C4RCD Research Group, Vincent Cantagrel, Karine Siquier-Pernet, Marlene Rio, Siddharth Banka, Ajoy Sarkar, Marcie Steeves, Michael Parker, Emma Clement, Sébastien Moutton, Frédéric Tran Mau-Them, Amélie Piton, Bert B.A. de Vries, Matthew Guille, Anne Debant, Susanne Schmidt, and Diana Baralle

A

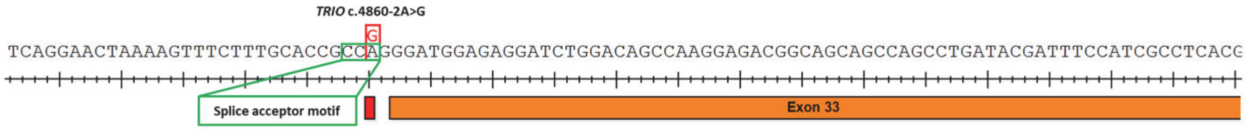

B

▾ HSF Matrices

Sequence Position	cDNA Position	Splice site type	Motif	New splice site	Wild Type	Mutant	If cryptic site use, exon length variation	Variation (%)
11	11	Acceptor	TTGCACCGCCAGGG	ttgcaccgccggGG	88.91	59.96	-11	Site broken -32.56
19	19	Acceptor	CCAGGGATGGAGAG	ccggggatggagAG	67.38	67.49	-19	+0.16
21	21	Acceptor	AGGGATGGAGAGGA	ggggatggagagGA	65.06	65.25	-21	+0.29

▾ MaxEnt

Threshold values:
5' Motif: 3 3' Motif: 3

Sequence Position	cDNA Position	5' Motif					3' Motif				
		Ref Motif	Ref Score	Mut Motif	Mut Score	Variation (%)	Ref Motif	Ref Score	Mut Motif	Mut Score	Variation (%)
3	3						AAGTTTCTTTGCACCGCCAGGGA	4.76	aagtttctttgcaccgccggGGA	-3.19	-167.02

C

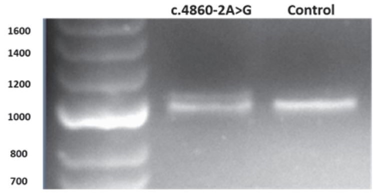

D

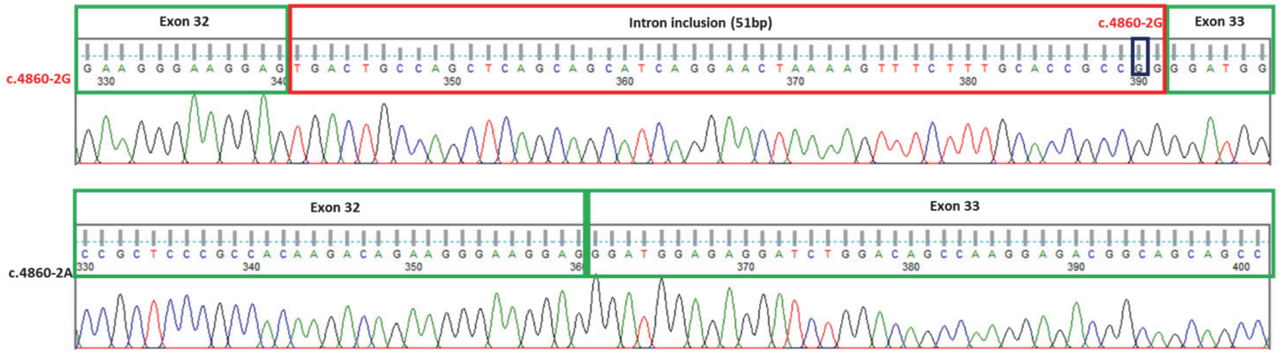

Figure S1: Impact of splice acceptor site variant in *TRIO* (c.4860-2A>G) on aberrant splicing. **A.** c.4860-2A>G is located in splice acceptor motif site of Exon 33. **B.** Human Splice Site Finder (HSF Version 3.1) and MaxEntScan predicted splice acceptor site broken. **C.** Gel electrophoresis shows an extra band in c.4860-2A>G PCR. **D.** Sanger sequencing shows an extra 51 nucleotides between Exon 32 and Exon 33.

Figure S1

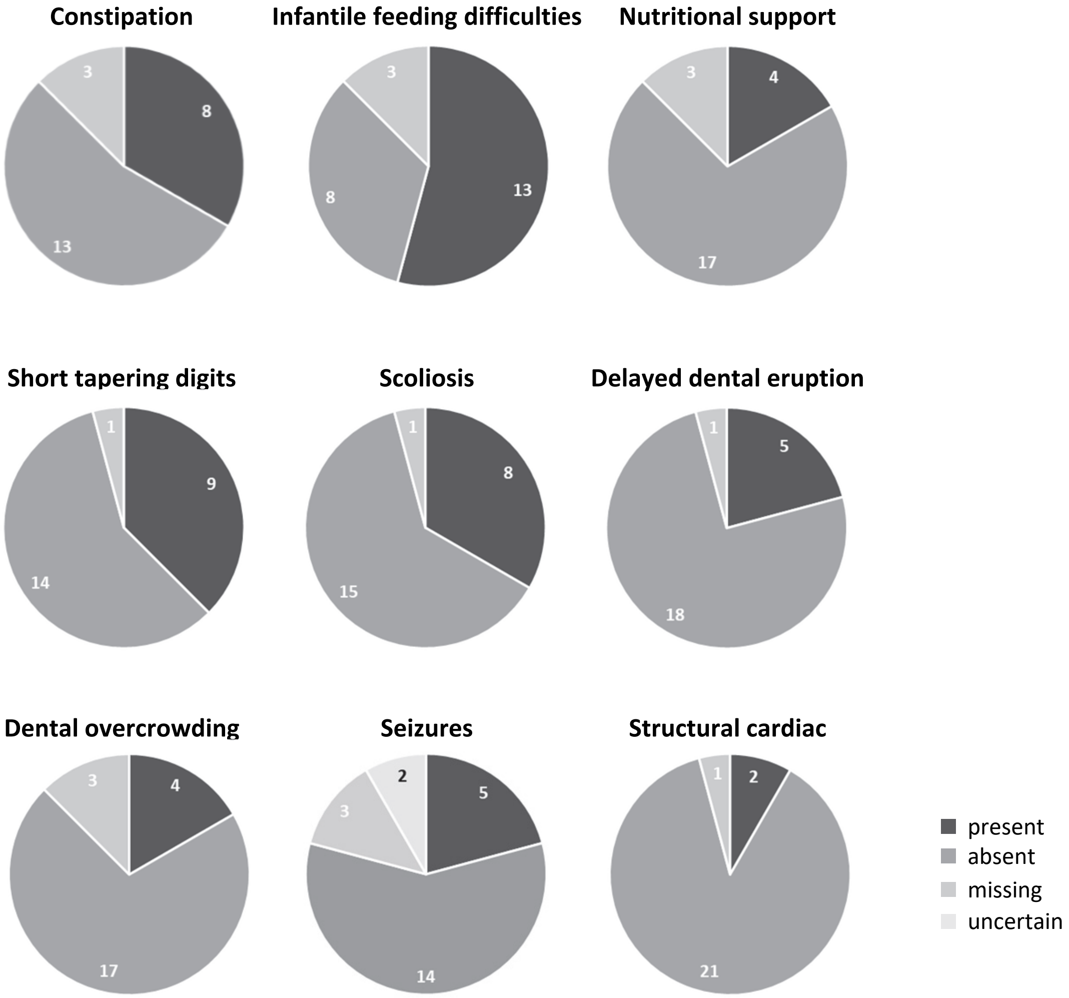

Figure S2: Expanded phenotype characteristics across the cohort. Gastrointestinal features including infantile feeding difficulties and constipation where frequently observed. Skeletal manifestations included short tapering digits, scoliosis and delayed dental eruption were also noted. Classified as: present, dark grey; absent, middle grey; missing, light grey and uncertain, very light grey.

A

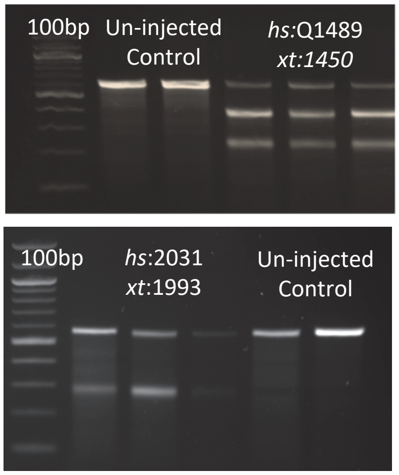

B

Head diameter (mm)			
	Control	Q1450	L1994
1	1.712	1.018	1.533
2	1.619	1.189	1.674
3	1.553	1.302	1.753
4	1.581	1.258	1.743
5	1.864	0.79	1.793
6	1.551	1.479	1.357
7	1.6	0.283	1.683
8	1.68	1.688	1.657

C

Control – Wild-type *Xenopus tropicalis*

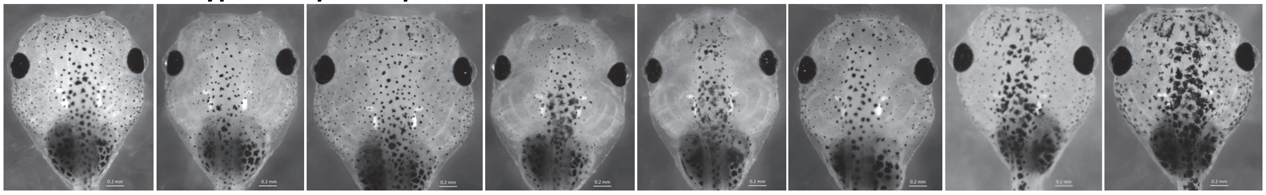

hs:Q1489, xt:Q1450 truncation

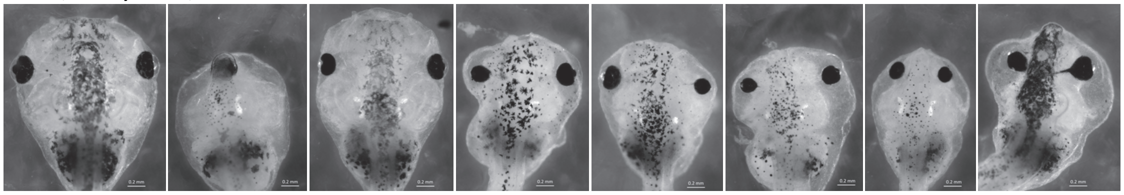

hs:L2031, xt:L1994 truncation

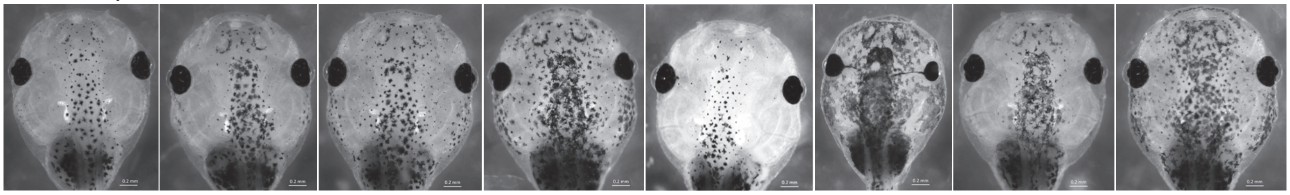

Figure S3: Truncation in the GEFD1 domain reduces head size in *X.tropicalis.*

A. Amplification of the target region followed by T7 endonuclease assay and Sanger sequencing confirmed that indels had been made at the target sites creating the truncations shown. **B.** Measurement of the head diameter of the injected tadpoles, 8 individuals per condition. **C.** Micrographs of all tadpoles used in the experiment.

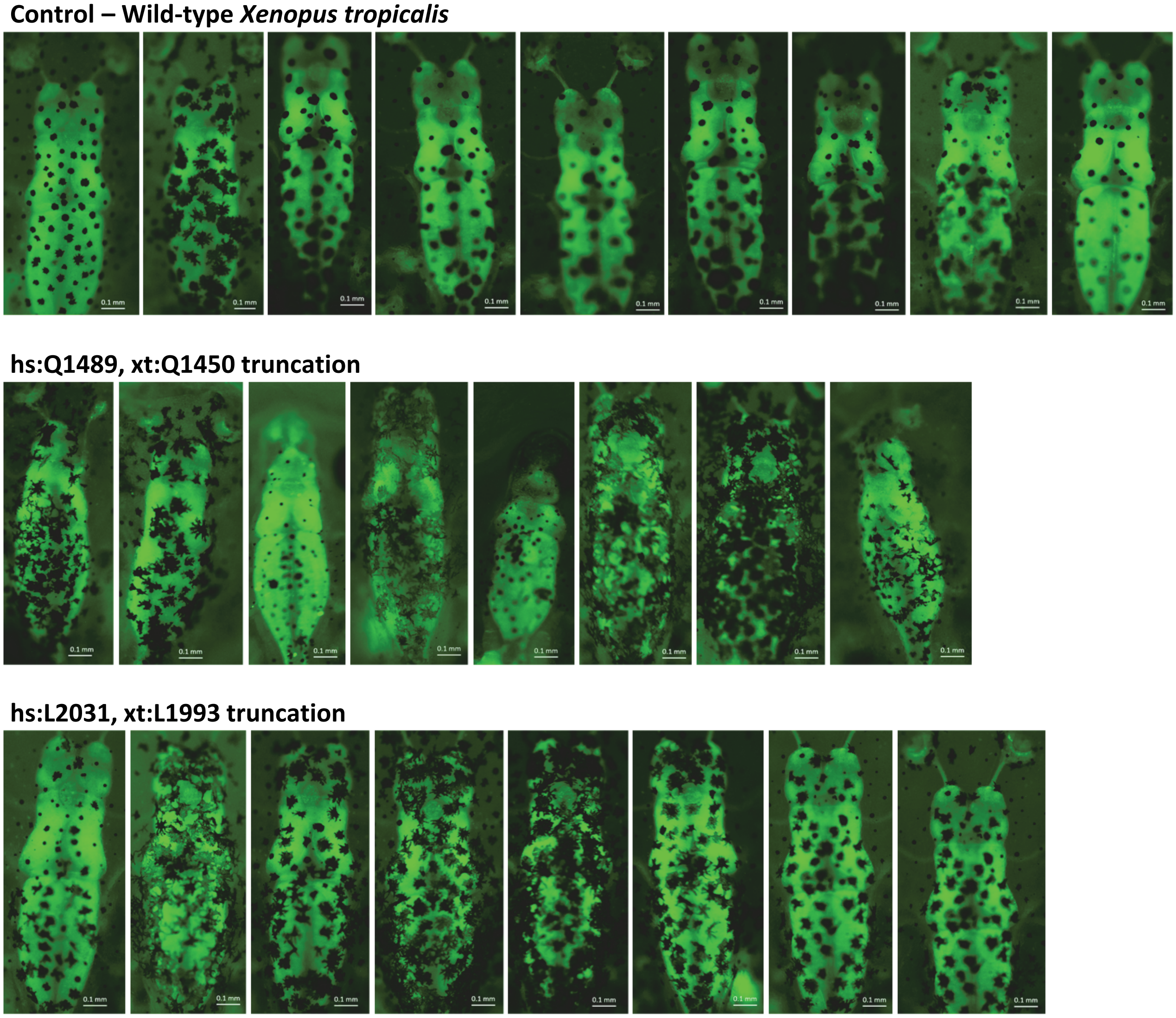

Figure S4: Truncation in the GEFD1 domain shows forebrain deformities in *X.tropicalis.*

GEFD1 and GEFD2 frameshifts were performed in a tubb2B.GFP transgenic *X. tropicalis* line, in order to examine gross structural abnormalities of the brain. This transgenic line labels differentiated neurons, and revealed forebrain deformities in the GEFD1 truncation.

**trio single guide RNAs (Human, Xenopus tropicalis)**	**Genotyping PCR primers**
Q1489*, Q1450	
taatacgactcactataGGTGAGAACATTGAATCTCAgttttagagctagaa	FWD: acaaacaactttaaaacccagaaaca
taatacgactcactataGGAAGAGTCTTTTCAAGTGTgttttagagctagaa	REV: ctgaaagcagctgagccagt
L2031*, L1993*	
taatacgactcactataGGTGAACTGGAAAGGTGCCgttttagagctagaa	FWD: atgctgcagaactgggattg
taatacgactcactataGGGTGAACTGGAAAGGTGCCgttttagagctagaa	REV: cctactcagcagggtcacag

Table S2: Oligonucleotides used to synthesise single guide RNAs and to amplify target regions of TRIO. Single guide RNA sequences were chosen using CRISPRscan; the target region itself is in uppercase and the oligonucleotides include a T7 RNA polymerase promoter in lowercase at the 5’ end and part of the conserved region that binds Cas9 in lowercase at the 3’ end. For the experiments shown here the upper sequence of each pair was used for both the GEFD1 and the GEFD2 truncating mutants.

	Age in Months						
	Domain	Min	1st Qu	Median	Mean	3rd Qu	Max	P value
First Words	Group 1	36	48	53	52	57	66	0.561
	Group 2	17	22.25	39	38.75	55.5	60	
Walking	Group 1	24	25.5	33	42	49.5	84	0.035
	Group 2	12	17	17	20.2	22	33	
Sitting	Group 1	7	9.75	11	17.36	24	36	0.168
	Group 2	8	9	9	9.33	9.75	11	
OFC	Group 1	53	54.3	56	55.98	57	59.5	<0.001
	Group 2	42	45	48	46.93	48.25	52	

Table S3: Statistical analysis of the developmental milestones of sitting, walking and speech, and ages achieved in either patient Group 1 of Group 2 mutation cases. Pearson's Chi-squared test was applied. Walking and OFC show a statistically significant difference between Group 1 and Group 2 individuals.
