# Supplementary material for: Opposite Modulation of RAC1 by Mutations in TRIO Is Associated with Distinct, Domain-Specific Neurodevelopmental Disorders
Source: Am J Hum Genet. 2020 Feb 27;106(3):338–55. doi: 10.1016/j.ajhg.2020.01.018 (PMC7058823; doi:10.1016/j.ajhg.2020.01.018)
Supplement: Document S2. Article plus Supplemental Information [file mmc3.pdf]

ARTICLE

Opposite Modulation of RAC1 by Mutations in *TRIO* Is Associated with Distinct, Domain-Specific Neurodevelopmental Disorders

Sónia Barbosa,[1,37] Stephanie Greville-Heygate,[2,37] Maxime Bonnet,[1] Annie Godwin,[4] Christine Fagotto-Kaufmann,[1] Andrey V. Kajava,[1] Damien Laouteouet,[1] Rebecca Mawby,[5] Htoo Aung Wai,[5] Alexander J.M. Dingemans,[6] Jayne Hehir-Kwa,[7] Marjorlaine Willems,[8] Yline Capri,[9] Sarju G. Mehta,[10] Helen Cox,[11] David Goudie,[12] Fleur Vansenne,[13] Peter Turnpenny,[14] Marie Vincent,[15] Benjamin Cogné,[15] Gaëtan Lesca,[16] Jozef Hertecant,[17] Diana Rodriguez,[18] Boris Keren,[19] Lydie Burglen,[20] Marion Gérard,[21] Audrey Putoux,[22] C4RCD Research Group,[23] Vincent Cantagrel,[24,25] Karine Siquier-Pernet,[25,26] Marlene Rio,[25,26] Siddharth Banka,[27,28] Ajoy Sarkar,[29] Marcie Steeves,[30] Michael Parker,[31] Emma Clement,[32] Sébastien Moutton,[33] Frédéric Tran Mau-Them,[34] Amélie Piton,[35,36] Bert B.A. de Vries,[6] Matthew Guille,[4] Anne Debant,[1,38] Susanne Schmidt,[1,38,*] and Diana Baralle[2,3,38,*]

The Rho-guanine nucleotide exchange factor (RhoGEF) TRIO acts as a key regulator of neuronal migration, axonal outgrowth, axon guidance, and synaptogenesis by activating the GTPase RAC1 and modulating actin cytoskeleton remodeling. Pathogenic variants in *TRIO* are associated with neurodevelopmental diseases, including intellectual disability (ID) and autism spectrum disorders (ASD). Here, we report the largest international cohort of 24 individuals with confirmed pathogenic missense or nonsense variants in *TRIO*. The nonsense mutations are spread along the *TRIO* sequence, and affected individuals show variable neurodevelopmental phenotypes. In contrast, missense variants cluster into two mutational hotspots in the *TRIO* sequence, one in the seventh spectrin repeat and one in the RAC1-activating GEFD1. Although all individuals in this cohort present with developmental delay and a neuro-behavioral phenotype, individuals with a pathogenic variant in the seventh spectrin repeat have a more severe ID associated with macrocephaly than do most individuals with GEFD1 variants, who display milder ID and microcephaly. Functional studies show that the spectrin and GEFD1 variants cause a TRIO-mediated hyper- or hypo-activation of RAC1, respectively, and we observe a striking correlation between RAC1 activation levels and the head size of the affected individuals. In addition, truncations in *TRIO* GEFD1 in the vertebrate model *X. tropicalis* induce defects that are concordant with the human phenotype. This work demonstrates distinct clinical and molecular disorders clustering in the GEFD1 and seventh spectrin repeat domains and highlights the importance of tight control of TRIO-RAC1 signaling in neuronal development.

[1]Centre de Recherche en Biologie Cellulaire de Montpellier, University of Montpellier, Centre National de la Recherche Scientifique 34293 Montpellier, France; [2]Wessex Clinical Genetics, University Hospital Southampton National Health Service Foundation Trust, Southampton SO16 5YA, UK; [3]Human Development and Health, Faculty of Medicine, University of Southampton, Southampton SO16 6YD, UK; [4]European Xenopus Resource Centre, School of Biological Sciences, University of Portsmouth, Portsmouth PO1 2DY, UK; [5]Human Development and Health, Faculty of Medicine, University of Southampton, Southampton SO16 6YD, UK; [6]Department of Human Genetics, Radboud University Medical Center, 6525 GA Nijmegen, the Netherlands; [7]Princess Máxima Center for Pediatric Oncology, 3584CS Utrecht, the Netherlands; [8]Département Génétique Médicale, Centre Hospitalier Régional et Universitaire de Montpellier, Montpellier 34295, France; [9]Département de Génétique, Centre Hospitalier Universitaire de Paris, Paris 75019, France; [10]Department of Clinical Genetics, Cambridge University Hospital Trust, Cambridge CB2 0QQ, UK; [11]West Midlands Regional Genetics Service, Birmingham Women's and Children's National Health Service Foundation Trust, Birmingham B15 2TG, UK; [12]Department of Clinical Genetics, Ninewells Hospital, Dundee DD2 1UB, UK; [13]Department of Clinical Genetics, University Medical Center, Groningen 9713 GZ Groningen, the Netherlands; [14]Clinical Genetics Department, Royal Devon and Exeter National Health Service Foundation Trust, Exeter EX1 2ED, UK; [15]Service de Génétique Médicale, Centre Hospitalier Universitaire de Nantes, 44093 Nantes, France; [16]Service de Génétique, Hospices Civils de Lyon, 69002 Lyon, France; [17]Tawam Hospital, PO Box 15258, Al Ain, United Arab Emirates; [18]Service de Neurologie Pédiatrique, Centre de Référence Maladies Rares – Neurogénétique, Sorbonne Université, Assistance Publique-Hôpitaux de Paris, Hôpital Armand Trousseau, 75012 Paris, France; [19]Département de Génétique (Pr Leguern), Hôpital Pitié-Salpêtrière, 75013 Paris, France; [20]Centre de Référence des Malformations et Maladies Congénitales du Cervelet, Département de Génétique et Embryologie Médicale, Hôpital Trousseau, 75012 Paris, France; [21]Service de Génétique, Centre Hospitalier Universitaire de Caen, Caen 14000, France; [22]Service de Génétique, Hospices Civils de Lyon, Bron 69500, France; [23]TGen's Center for Rare Childhood Disorders, Translational Genomics Research Institute, Phoenix, AZ 85012, USA; [24]Paris Descartes-Sorbonne Paris Cite University, Imagine Institute, 75015 Paris, France; [25]Developmental Brain Disorders Laboratory, INSERM UMR 1163, 75015 Paris, France; [26]Service de Génétique, Necker Enfants Malades University Hospital, Assistance Publique-Hôpitaux de Pairs, 75015 Paris, France; [27]Division of Evolution and Genomic Sciences, School of Biological Sciences, Faculty of Biology, Medicine and Health, University of Manchester, Manchester M13 9WL, UK; [28]Manchester Centre for Genomic Medicine, St Mary's Hospital, Manchester University NHS Foundation Trust, Health Innovation Manchester, Manchester M13 9WL, UK; [29]Department of Clinical Genetics, Nottingham University Hospitals National Health Service Trust, Nottingham NG5 1PB, UK; [30]Mass General Hospital for Children, Boston, MA 02114, USA; [31]Clinical Genetics, Sheffield Children's National Health Service Foundation Trust, Sheffield S10 2TH, UK; [32]Clinical Genetics Department, Great Ormond Street Hospital for Children National Health Service Foundation Trust, London WC1N 3JH, UK; [33]Reference Center for Developmental Anomalies, Department of Medical Genetics, Dijon University Hospital, 21000 Dijon, France; [34]INSERM U1231, Service de Génétique des Anomalies du Développement, Burgundy University, F-21000 Dijon, France; [35]Institut de Génétique et de Biologie Moléculaire et Cellulaire, Strasbourg, University of Strasbourg, 67404 Illkirch, France; [36]Laboratory of Genetic Diagnostic, Hôpitaux Universitaires de Strasbourg, 67091 Strasbourg, France
[37]These authors contributed equally
[38]These authors contributed equally
*Correspondence: susanne.schmidt@crbm.cnrs.fr (S.S.), d.baralle@soton.ac.uk (D.B.)
https://doi.org/10.1016/j.ajhg.2020.01.018.
© 2020 The Authors. This is an open access article under the CC BY license (http://creativecommons.org/licenses/by/4.0/).

Introduction

The Rho guanosine triphosphatases (GTPases) play an essential role in many neurodevelopmental steps, including neurogenesis, migration, and the formation of synapses, by regulating actin cytoskeleton dynamics.[1] They are activated by guanine nucleotide exchange factors (GEFs) that catalyze guanosine diphosphate (GDP) dissociation and allow the binding of guanosine triphosphate (GTP). By facilitating this GDP-to-GTP exchange, GEFs act as intermediaries between an external cue and the activation of Rho GTPases.[2] TRIO is a highly conserved GEF that contains two GEF domains and numerous accessory motifs, including spectrin repeats at its N terminus.[3] TRIO is a member of a small Rho-guanine nucleotide exchange factor (RhoGEF) sub-family that includes two functional GEF domains. The first GEF domain (GEFD1) regulates RAC1 and RHOG activity, and the second GEF domain (GEFD2) regulates RHOA activity.[4,5] It is recognized that through RAC1 activation and actin cytoskeleton remodeling, TRIO acts as a major regulator of cytokinesis, cell migration, axon outgrowth, axon guidance, and dendritic arborization and plays a role in synaptogenesis by modulating excitatory synaptic transmission.[6–10]

The importance of *TRIO* in development is demonstrated in murine models. *TRIO* knockout in mice is embryonically lethal, and embryos show abnormalities in skeletal muscle and neural-tissue development.[11] Mice in which *TRIO* has been specifically deleted in the nervous system die perinatally, and the few mice that survive display defects in the migration of cerebellar granule cells.[12] Heterozygous or homozygous deletion of *TRIO* in the hippocampus and in the cortex during early embryogenesis results in progressive defects in learning ability, sociability, and motor coordination of these mice.[13–15]

Whole-exome sequencing studies have identified several deleterious *de novo* mutations in *TRIO* in different cohorts of individuals with neurodevelopmental disorders, including intellectual disability (ID) and/or autism spectrum disorders (ASD).[16–19] In addition, *TRIO* has been reported as intolerant to functional genetic variation.[20] The reported clinical phenotypes of affected individuals with *TRIO* mutations include intellectual disability, behavioral difficulties such as hyperactivity or aggression, autism or autistic behavioral tendencies, skeletal hand anomalies, and microcephaly. Pathogenic variants include missense mutations and nonsense mutations, the latter of which generates premature stop codons leading to truncated proteins or haploinsufficiency. Missense mutations in *TRIO* often target TRIO's RAC1-activating domain, GEFD1. For the GEFD1 mutants on which there have been functional studies, the majority show a reduction in TRIO-mediated activation of RAC1,[17–19] but some also affect glutamatergic synaptic transmission, reinforcing the hypothesis that *TRIO* mutations are causative of these neurodevelopmental disorders.[18]

In addition to this cluster of missense mutations targeting the GEFD1, we have previously described an individual harboring a missense mutation in one of the spectrin motifs.[17] In comparison to the severe microcephaly seen in individuals with mutations in the GEFD1, this person had a head circumference in the 75th centile.[17] This suggests that the position of the mutation in the *TRIO* sequence could differentially impact TRIO's neuronal function and cause phenotypic heterogeneity.

Here, we report on an important set of individuals harboring pathogenic *TRIO* (MIM: 601893) variants, including nonsense and missense mutations. The nonsense mutations are spread along the *TRIO* sequence, and individuals show variable neurodevelopmental phenotypes. With regard to the missense mutations, we have identified the seventh spectrin domain of *TRIO* as a second mutational hotspot and describe how this gives rise to a distinct phenotype of severe neurodevelopmental delay, macrocephaly, and TRIO-mediated RAC1 hyperactivity. This is in contrast to the GEFD1 hotspot, which is mostly associated with milder ID, microcephaly, and reduced TRIO activity. In addition, expression of *TRIO* variants in the vertebrate model *X. tropicalis* induces defects that are concordant with the human phenotype, showing that *TRIO* mutations are causative of these neuordevelopmental disorders. Altogether, we propose that, depending on the domain targeted, mutations affect TRIO's function in opposite ways and give rise to two seemingly diverse clinical syndromes: severe developmental delay and macrocephaly (spectrin variants) versus a milder developmental phenotype and microcephaly (GEFD1 variants). These findings highlight the importance of tight control of TRIO-RAC1 signaling during neuronal development.

Material and Methods

Identification of Pathogenic *TRIO* Variants

Pathogenic variants in *TRIO* were identified by whole-exome sequencing performed on whole blood DNA as part of the Deciphering Developmental Disorders (DDD) Research Study,[21] as part of a research study at TGen WIRB Protocol #20120789, Genomics England 100,000 Genomes Project (see The National Genomics Research and Healthcare Knowledgebase v5 in Web Resources), or through diagnostic clinical practice. The DDD study collated more than 12,000 affected individuals with undiagnosed neurodevelopmental disorders. Variants were reported according to standardized nomenclature defined by the reference human genome GRCh37 (hg19) and *TRIO* transcript GenBank: NM_007118. The minor-allele frequency of each variant was determined from genomic sequencing data derived from the gnomAD, and the effect of each genomic variant on protein function was predicted with the Ensembl Variant Effect Predictor.[22] None of the missense variants are listed in the gnomAD.

Aberrant Splicing Analysis

Blood was collected from individual 19, and RNA was extracted from the blood sample with the PAXgene Blood RNA Kit (PreAnalytiX). cDNA synthesis was performed with the High

Capacity cDNA Reverse Transcription Kit (Thermo Fisher Scientific). The *TRIO* variant (c. 4860−2A>G) is located on the intronic splice acceptor site of exon 33 (GenBank: NM_007118.4), and the primer pairs (pair 1 [forward] 5′-AGTGGGAGAAGCAAGTACCT-3′/[reverse] 5′-CTGTCGTCGGAGTCCTTCTG-3′ and pair 2 [forward] 5′-AGAACGGCATCTCTTCCTTTT-′/[reverse] 5′-CACTGTCGTCGGAGTCCTT-3′) (Integrated DNA Technologies) were designed on the flanking exon 30 and exon 35. PCR was performed with the GoTaq G2 Polymerase PCR system (Promega). The amplicons were visualized under ChemiDoc XRS+ (Bio-Rad). Amplicons for further analysis were cloned into plasmids via a TA cloning kit with the pCR 2.1 vector (Thermo Fisher Scientific). Plasmids carrying inserts were sequenced by Sanger sequencing (Source Bioscience). Bioinformatic predictions for aberrant splicing were performed with Human Splicing Finder (version 3.1),[23] MaxEntScan,[24] and Splice Site Finder by Neural Network (SSFNN).[25]

Patient Consent

Patient consent for participation and phenotyping was obtained through the referring clinical team. Referring clinicians were requested to complete a comprehensive phenotyping questionnaire that was based upon our current understanding of the phenotypic associations of *TRIO*. This included sections related to neurodevelopmental screening; dysmorphology; and gastrointestinal, skeletal, and cardiac phenotypic features. Consent and collection of information conformed to the recognized standards of the Declaration of Helsinki.

Quantitative Facial Phenotyping

Clinical photographs of individuals' faces were analyzed according to the hybrid model reported previously.[26,27] This model combines two algorithms (OpenFace[28] and Clinical Face Phenotype Space[29]) used for facial recognition to create a 468-dimensional vector of the facial features of a given individual. These vectors are used for calculating the clustering impact factor (CIF) of a patient group. This is a measurement of how a group of individuals cluster within a group of controls; these controls are age-, ethnicity-, and gender-matched individuals with intellectual disability (see van der Donk et al.[26] for further technical details). The Mann-Whitney U test is utilized for determining whether the CIF is significantly higher than expected on the basis of random chance. This was done for all individuals, including the subset of individuals with mutations in either of the domains (GEFD1 and spectrin), so that the facial features of individuals with mutations in these different domains could be assessed for differences. A p value smaller than 0.0125 (0.05/4) was considered significant, after correcting for multiple testing.

Molecular Modeling of TRIO Domains

We modeled the structure of the seventh spectrin repeat of TRIO on the basis of the crystal structures of human beta2-spectrin (PDB ID 3EDV[30]) by using a sequence alignment obtained from the generalized sequence profile.[31] The model was constructed by the SWISS-MODEL server.[32] The structure of the DH1 domain of TRIO in complex with the small GTPase substrate RAC1 was modeled with the crystal structures of DH1 (PDB: 1NTY) and the complex with substrate (PDB: 1KZ7). Data for the protein structures were generated with PyMOL.[33]

Plasmids and DNA Constructs

The pbio-EGFP-TRIO and pbio-EGFP-TRIO GEF-dead constructs have been described previously.[7,34] All the missense and nonsense mutants were generated with the QuikChange Site-directed mutagenesis kit (Agilent Technologies) according to the manufacturer's instructions; appropriate primers were used. Primer sequences are available upon request. All constructs were verified by sequencing. pLXSN-myc-Rac1^{T17N} has been described previously.[35] The pRK5-Myc-PAK1 construct was a kind gift from Nathalie Lamarche-Vane (McGill Cancer Research Center, Montreal, Canada).

Cell Culture and Transfection

N1E-115 and HEK293T cells were cultured at 37°C in Dulbecco's modified Eagle's medium (DMEM; Eurobio) supplemented with 10% fetal bovine serum, 2 mM L-glutamine (Invitrogen), and penicillin and streptomycin (Invitrogen) under humidified conditions with 5% CO_2.

For the neurite outgrowth assays, N1E-115 cells were seeded in 35 mm dishes containing 12 mm glass coverslips coated with laminin (25 μg/mL; Sigma Aldrich). HEK293T cells were seeded in 100 mm dishes. Cells were transfected with TRIO constructs as indicated with the JetPEI reagent (Polyplus) at 1:5 (for N1E-115 cells) and 1:3 (for HEK293T cells) ratios, as described previously.[36]

RAC1^{N17} Binding Assay

HEK293T cells were co-transfected with the indicated biotinylated pbioGFP-TRIO variants and a pLXSN-Myc-RAC1^{N17} plasmid. 48 h after transfection, cells were lysed in lysis buffer [50 mM Tris HCl (pH 7.5), 1 mM EGTA, 1 mM EDTA, 0.1% (w/v) Triton X-100, 1 mM sodium orthovanadate, 50 mM sodium fluoride, 5 mM sodium pyrophosphate, 10% sucrose, 1 mM dithiothreitol, 0.5 mM 4-(2-aminoethyl)benzenesulfonyl fluoride hydrochloride (AEBSF)]. TRIO was pulled down with Streptavidin Dynabeads (Invitrogen), and the co-precipitating RAC1^{N17} was detected via immunoblotting with a RAC1 antibody (BD Biosciences, #610651). Total cell lysates were analyzed with the relevant anti-RAC1 and anti-GFP antibody (Torrey Pines Biolabs, #TP401).

Neurite Outgrowth and Lamellipodia Analysis

More than 200 transfected N1E-115 cells were analyzed for each condition. A neurite was defined as a process that measured at least twice the length of the cell body. Lamellipodia were defined as sheet-like rhodamine-phalloidin-positive protrusions, occurring along or at the tip of the neurite. The proportion of transfected cells harboring a neurite or a lamellipodium was determined manually in an experimenter-blinded procedure. Values obtained from no less than five independent experiments were presented as n-fold change over wild-type (WT) TRIO, which was arbitrarily set to 1. A Student's unpaired t test was used for statistical analysis.

Immunoblot Analysis of Phospho-PAK Amounts

HEK293T cells were co-transfected with the indicated pbioGFP-TRIO variants and a pRK5-PAK1 plasmid. 48 h after transfection, cells were lysed in lysis buffer, and amounts of phosphorylated PAK1 were monitored by immunoblot analysis with a phospho-Ser144 PAK antibody (# 2606S, Cell Signaling) and a monoclonal PAK1 antibody (sc-166887). Total TRIO expression was detected with a GFP antibody (Torrey Pines Biolabs, #TP401). Immunoblot detections and band-intensity quantifications were made with the Odyssey system from Li-COR Biosciences.

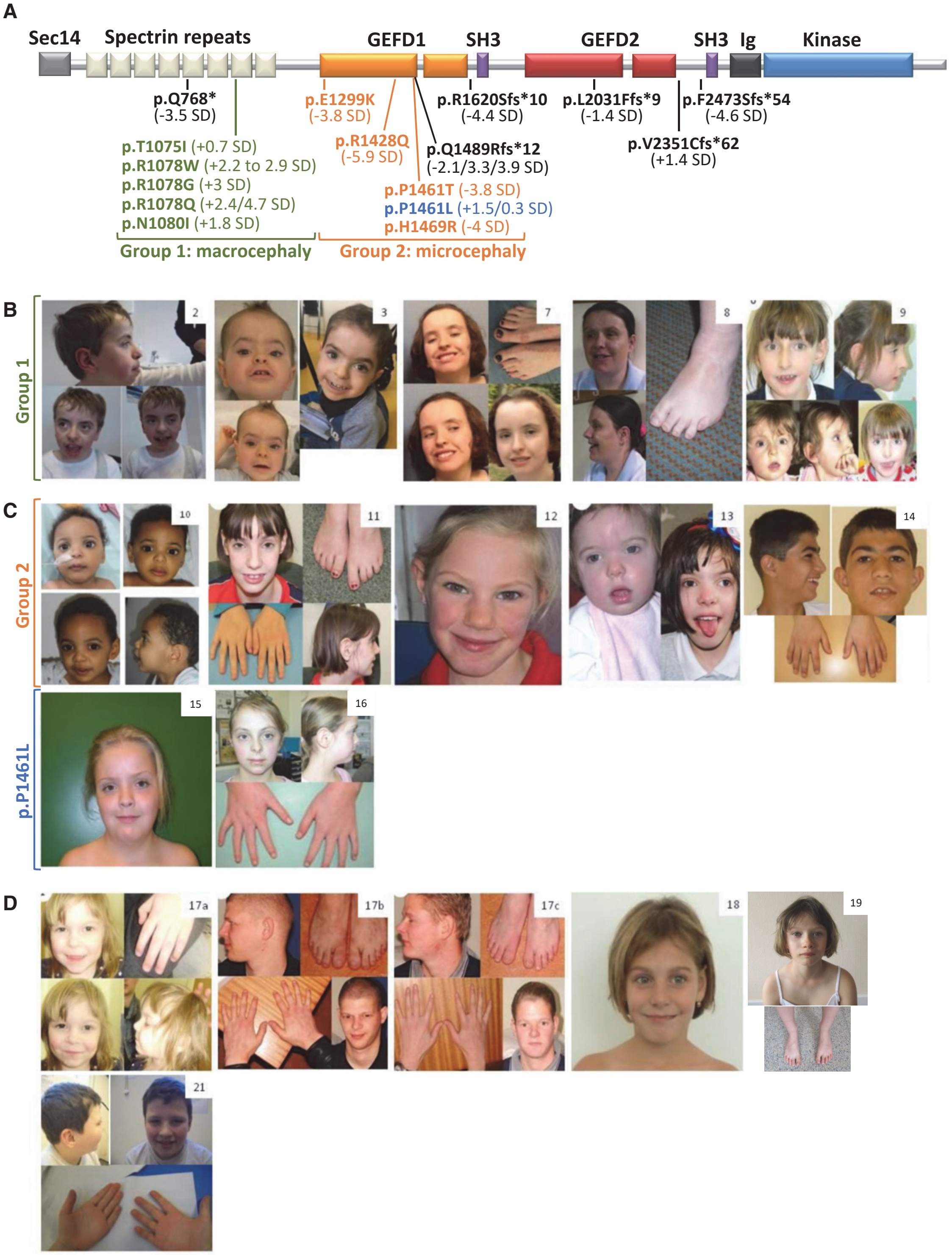

Figure 1. Pathogenic *TRIO* Variants Found in Individuals with Neurodevelopmental Disorders

(A) Schematic representation of TRIO domains with annotated missense and nonsense mutations identified in affected individuals. Patient groups 1 and 2 are highlighted in green and orange, respectively. Missense mutation p.Pro1461Leu is in blue so that it is differentiated from the other GEFD1 mutants (see text for details). Nonsense variants are written in black. The numbers in brackets correspond to the OFC of each affected individual (+/−SD).

(B) Clinical photographs of individuals carrying an alteration in the seventh spectrin repeat domain of TRIO (group 1). Individuals 2 and 3 have the p.Arg1078Trp variant, individuals 7 and 8 have the p.Arg1078Gln variant, and individual 9 has the p.Asn1080Ile variant.[17]

(legend continued on next page)

Animal Maintenance and Embryo Generation at the European *Xenopus* Resource Center

All work presented was conducted in accordance with the conditions set out by the UK's Home Office legislation under PPL 70/6450 (Professor Matthew Guille: European *Xenopus* Resource Centre) and approved by the University of Portsmouth's Animal Welfare Ethical Review Body. Outbred Nigerian *Xenopus tropicalis* were maintained in recirculating systems with 15% daily water changes and temperatures between 25°C–27°C and maintained on a 13–11 h light-dark cycle. Adult *X. tropicalis* were fed thrice daily five days per week with Skretting Horizon 23 pellets. Sexually mature female *X. tropicalis* were primed the evening before collection with 10 IU hCG and a boosting dose of 100 IU hCG the following morning. Egg clutches were obtained via gentle abdominal massage and fertilized with cryopreserved sperm.

Frozen *X. tropicalis* spermatozoa were generated from adult male *X. tropicalis* exhibiting enhanced nuptial pads. Sperm cryopreservation followed an adapted protocol initially developed by Sargent and Mohun[37] and modified by Mansour[38] with further amendments implemented within the European *Xenopus* Resource Centre.[39] Cryopreserved spermatozoa were rapidly thawed in a 37°C water bath. Two volumes of 0.1 × Marc's Modified Ringer's Solution (MMR: 0.01 M NaCl, 0.2 mM KCl, 0.1 mM $MgSO_4$, 0.2 mM $CaCl_2$, and 0.5 mM HEPES [pH 7.4]) was then added to the thawed spermatozoa, and the solution was applied directly to eggs manually spread to a monolayer. Embryos were maintained in 0.05 × MMR (containing penicillin [5 U/mL] and streptomycin [5 μg/mL]) with 50% daily media changes at 25.5°C in 145 mm tissue-culture Petri dishes at a density of 50 embryos (or fewer) per dish.

Use of CRISPR-Cas for Generation of Knock-Out Animals

Xenopus tropicalis target regions of interest were identified through Xenbase, and four single-stranded oligonucleotides (Table S2) directed at multiple loci within *TRIO* were designed with CRISPRscan. Subsequently, single-guide RNAs were generated according to the PCR-based method described by Nakayama and colleagues.[40] *X. tropicalis* embryos were co-injected at the one-cell-stage with 500 pg single-guide RNA and 2.5 μg Cas9 (spy Cas9 NLS, NEB) with the microinjection apparatus and procedure described in Guille.[41] Genomic DNA was extracted from individual embryos with QIAGEN's DNeasy Blood and Tissue Kit. Primers (Table S2), designed in Primer3, were used for amplification of the target regions of interest for analysis. T7 Endonuclease I (NEB), which recognizes and cleaves mismatched DNA, was used for identification of the most effective single-guide RNA for each site.[42] Sanger sequencing of PCR-amplified regions confirmed these indels in mutant embryos. Tadpoles were imaged with a ZEISS Axio Zoom.V16 Stereomicroscope. All analyses were implemented with packages in R version 3.5.1. Differences in head diameter between treatments were analyzed via a one-way analysis of variance (ANOVA). A Tukey honestly significant difference (HSD) post-hoc test was used.

Results

Cohort of Individuals with Pathogenic Variants in *TRIO*

Here, we present the largest phenotypic cohort of individuals who had confirmed pathogenic variants in *TRIO* and who presented with neurodevelopmental delay. Through a combination of DDD and internationally ascertained participants, we recruited 24 individuals from 22 families. Pathogenic *TRIO* variants were *de novo* in 21 families and inherited in one family. The mechanism of inheritance was not described in three families. Different variant types were observed: 16 missense variants (nine within the N-terminal spectrin repeat domain and seven within the GEFD1), seven nonsense (protein-truncating) variants, and one consensus splice-site variant (Figure 1A, Table 1).

Missense mutations were clustered into two main groups: the seventh spectrin repeat domain of *TRIO* (group 1) and the GEFD1 (group 2) (Figure 1 A). Individuals 1 to 9 harbor missense mutations in the seventh spectrin repeat at the N terminus of *TRIO* (Figure 1A, Tables 1 and S1). Interestingly, these variants cluster within only three amino acids (Thr 1075, Arg 1078, and Asn 1080). Within group 1, we demonstrate a consistent phenotype of severe intellectual disability and macrocephaly (Figure 1B, Tables 1 and S1). Individuals 10 to 16 carry missense mutations in residues within the RAC1-activating GEFD1; such residues include two amino acids (Arg 1428 and Pro 1461) mutated in several unrelated patients (Figure 1A). These individuals, except individuals 15 and 16, define patient group 2 and present with milder ID and microcephaly. Individuals 15 and 16 (harboring the p.Pro1461Leu mutation) were excluded from group 2 in our analysis because they presented no microcephaly (Figure 1C, Tables 1 and S1).

A third group of persons, individuals 17 to 22, carry nonsense mutations throughout the *TRIO* sequence but have no specific mutational cluster and show a more variable neurodevelopmental phenotype (Tables 1 and S1, Figures 1A and 1D). These nonsense mutations presumably lead to the formation of a truncated protein, featuring only parts of the functional domains of TRIO. Individuals 17a–17c carry an inherited nonsense GEFD1 mutation, p.Gln1489Argfs*12 (reported previously as p.Gln1489Argfs*11),[17] generating a truncated and thus non-functional GEFD1. Interestingly, they present with a phenotype similar to that of group 2 patients. Alternatively, the frameshift mutants, and especially mutant p.Gln768* at the N terminus of *TRIO*, could be subject to nonsense-mediated decay of the truncated transcript; such decay could result in negligible protein production and haploinsufficiency. Of interest, individual 19 carries

(C) Clinical photographs of individuals carrying an alteration in the GEFD1 of TRIO (group 2 and the p.Pro1461Leu variant). Individuals 10–16 harbor the following variants: (10) p.Glu1299Lys, (11 and 12) p.Arg1428Gln, (13) p.Pro1461Thr,[17] (14) p.His1469Arg, (15 and 16) p.Pro1461Leu.

(D) Clinical photographs of individuals carrying nonsense mutations: individuals 17a–17c all carry the p.Gln1489Argfs*12 variant and are related;[17] patient 17a is the daughter of individual 17b. Individuals 17b and 17c are brothers. Individuals 18, 19, and 21 carry the nonsense variants p.Gln768*, p.Arg1620Serfs*10, and p.Val2351Cysfs*62, respectively.

Table 1. Individual Clinical Phenotype

	Group 1 Individuals									Group 2 Individuals		
**Patient**	**1**	**2**	**3**	**4**	**5**	**6**	**7**	**8**	**9**	**10**	**11**	**12**
**TRIO variant**												
Coding change	c.3224C>T	c.3232C>T	c.3232C>T	c.3232C>T	c.3232C>T	c.3232C>G	c.3233G>A	c.3233G>A	c.3239A>T	c.3895G>A	c.4283G>A	c.4283G>A
Protein change	p.Thr1075Ile	p.Arg1078Trp	p.Arg1078Trp	p.Arg1078Trp	p.Arg1078Trp	p.Arg1078Gly	p.Arg1078Gln	p.Arg1078Gln	p.Asn1080Ile	p.Glu1299Lys	p.Arg1428Gln	p.Arg1428Gln
Domain	spectrin	spectrin	spectrin	spectrin	spectrin	spectrin	spectrin	spectrin	spectrin	GEFD1	GEFD1	GEFD1
Inheritance	NR	*de novo*	*de novo*	*de novo*	*de novo*	*de novo*	*de novo*	*de novo*	*de novo*	*de novo*	*de novo*	*de novo*
Sex	M	M	M	M	M	M	F	F	F	M	F	F
Age last assessment	15 years	12 years	3 years	4 years	7 years	6 years	19 years	40 years	9 years	20 months	16 years	8 years
First smile	NR	NR	<36 weeks	52 weeks	NR	normal	NR	24 weeks	NR	12 weeks	8 weeks	6 weeks
Sitting unsupported	12 months	NR	NA	36 months	36 months	7 months	10.5 months	9 months	11 months	9 months	10 months	8 months
Walking unaided	30 months	84 months	NA, crawling	NA	NA	24 months	24 months	36 months	48–60 months	NA	22 months	12 months
First words	66 months	NR	NA	NA	36 months	NA	48–60 months	52 months	NR	24 months	NR	NR
Learning Difficulties	yes; moderate	yes; severe	yes; severe	yes; severe	yes; severe	yes; severe	yes; moderate	yes; severe	yes; severe	yes; moderate	missing level in charts	yes; moderate
Language delay: Expression	yes; dysarthria, short sentences	yes; 50 words at 7 years	yes; babble only	yes; babble only	yes; <10 words	yes; severe, sounds only	yes; impaired articulation	yes; short sentences only	yes; non-verbal, uses Makaton	yes; only a few two- syllable words	yes; very talkative	yes; talkative
Language delay: Comprehension	yes	yes	yes	yes	yes	yes; comprehension better	yes	yes	yes	yes	yes; unable to read or write	yes
OFC	+0.7 SD	+2.9 SD	+2.2 SD	+2.7 SD	+2.8 SD	+3 SD	+2.4 SD	+4.7 SD	+1.8 SD	−3.8 SD	−5.9 SD	"microcephaly"
Other genetic variants	NR	14q21.1 microdeletion	no	no	no	NR	no	NR	no	no	no	maternally inherited 4p microdeletion
Reference (patient)	this study	this study	this study	this study	this study	this study	this study	this study	17	this study	17	this study
Variant described in	-	-	-	-	-	-	-	-	17	-	17, 18	17, 18

(*Continued on next page*)

Table 1. *Continued*

	Group 2 Individuals				Truncation Individuals							
**Patient**	**13**	**14**	**15**	**16**	**17a (proband)**	**17b (father)**	**17c (uncle)**	**18**	**19**	**20**	**21**	**22**
**TRIO Variant**												
Coding change	c.4381C>A	c.4406A>G	c.4382C>T	c.4382C>T	c.4466delA	c.4466delA	c.4466delA	c.2302C>T	c.4860−2A>G	c.6092dup	c.7050del	c.7461del
Protein change	p.Pro1461Thr	p.His1469Arg	p.Pro1461Leu	p.Pro1461Leu	p.Gln1489Argfs* 12	p.Gln1489Argfs* 12	p.Gln1489Argfs* 12	p.Gln768*	p.Arg1620Serfs* 10	p.Leu2031Phefs*9	p.Val2351Cysfs* 62	p.Phe2473Serfs* 54
Domain	GEFD1	GEFD1	GEFD1	GEFD1	truncating	truncating	truncating	truncating	truncating	truncating	truncating	truncating
Inheritance	*de novo*	*de novo*	*de novo*	*de novo*	inherited	inherited	inherited	NR	*de novo*	*de novo*	*de novo*	NR
Sex	F	M	F	F	F	M	M	F	F	M	M	M
Age last assessment	8 years	14 years	19 years	11 years	17 months	36 years	10 years	14 years	8 years	16 years	9 years	21 months
First smile	36 weeks	NR	8 weeks	28 weeks	NR	NR	NR	NR	NR	6 weeks	>8 weeks	21 months
Sitting unsupported	11 months	9 months	11 months	13 months	9 months	NR	NR	NR	8 months	6 months	NR	NA
Walking unaided	30–36 months	17 months	21 months	24 months	17 months	NR	NR	14 months	12 months	12 months	>24 months	NA
First words	48–60 months	60 months	NR	24–36 months	17 months	NR	NR	24 months	10 months	Delayed	>18 months	NA
Learning difficulties	yes; global	yes; mild	yes; mild	yes; moderate	yes; mild	yes; mild	yes; mild	yes	yes; mild	yes; severe	yes; moderate	yes; severe
Language delay: expression	yes	yes; first sentences at 9 years	yes	yes; literal speech	yes	NR	NR	yes	yes	yes; short sentences	yes	yes; no language development
Language delay: comprehension	yes	no	yes	yes	yes	NR	NR	yes	yes	no	yes	yes
OFC	−3.8 SD	−4 SD	+1.5 SD	0.3 SD	−3.3 SD	−2.1 SD	−3.9 SD	−3.5 SD	−4.4 SD	−1.4 SD	+1.4 SD	−4.6 SD
Other genetic variants	-	-	no	bi-allelic FAT4 variants regarded as VUS	no	15q11.2 microdeletion and maternal DM	15q11.2 microdeletion	no	no	no	5p15.31 VUS paternally inherited, father phenotypically normal	no
Reference (patient)	17	this study	this study	this study	17	17	17	this study	this study	this study	this study	this study
Variant described in	17, 18	-	18	18	-	-	-	-	-	-	-	-

Description of the clinical phenotype observed among *TRIO* mutation carriers, grouped on the basis of variant location or protein effect: spectrin repeat (group 1), GEFD1 (group 2 and p.Pro1461Leu individuals 15 and 16) and protein truncating. Abbreviations are as follows: M, male; F, Female; NR, not recorded; SD, standard deviation; OFC, occipitofrontal circumference; GORD, gastro-esophageal reflux disease; NG, nasogastric; and NA, not achieved.

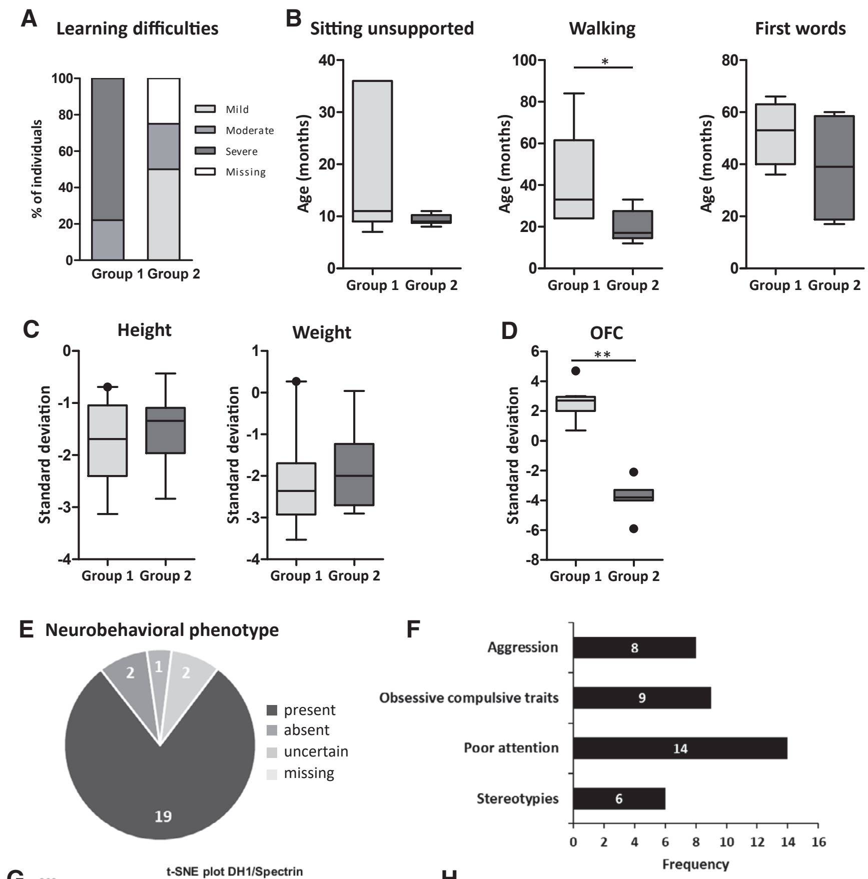

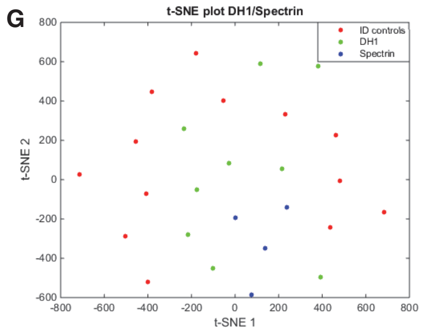

H

Patient group	CIF	P value
All patients (n=13)	0.8925	0.44146
GEFD1 (n=9)	0.9463	0.44506
Spectrin (n=4)	3.2143	0.01199
GEFD1 (n=9) vs Spectrin (n=4)	2.42	0.05559

Figure 2. Individuals Harboring Variants in Either GEFD1 or Spectrin Domains Show Distinct Neurodevelopmental Phenotypes

(A) Percentage of affected individuals with different levels of learning difficulties in either spectrin domain (group 1) or GEFD1 (group 2) mutation cases. 75% of group 2 individuals have mild-moderate learning difficulties. 78% of group 1 individuals have severe learning difficulties.

(B) Early developmental milestones (sitting unsupported, walking, and first words) are delayed in both group 1 and group 2 individuals, but group 1 individuals are affected more severely. For statistical analysis, refer to Table S3. Walking: *$p = 0.035$.

(C) Height and weight standard deviations from mean in either group 1 or group 2 individuals. The median height for group 1 individuals with spectrin mutations was −1.69 SD, and for group 2 individuals with GEFD1 mutations it was −1.34 SD. 33% of the individuals in group 1 and 14% of individuals in group 2 had a height SD of greater than −2 SD, which is often considered as short stature. Weight SD was also reduced in both groups.

(D) Microcephaly was seen in 100% of group 2 individuals, who had a mean OFC of −3.82 SD (median OFC of −3.8 SD). Individuals within group 1 present with macrocephaly. 78% had an OFC greater than 2 SD from the mean; mean OFC was +2.6 SD, median OFC was + 2.7 SD, and OFC range was between +0.7 SD and +4.7 SD. **$p < 0.001$ (Table S3)

(E) A neurobehavioral phenotype was observed in 19 out of the 24 (79%) individuals described in this study.

(F) Recurrent behavioral features include stereotypies (6/24 patients), poor attention (14/24 individuals), obsessive-compulsive traits (9/24 individuals), and aggression (8/24 individuals). In total, 2/24 were identified as social or friendly.

(G and H) Quantitative facial phenotyping. t-distributed stochastic neighbor embedding (t-SNE) plot of the vectors of individuals with alterations in GEFD1 and the seventh spectrin domain and the matched controls with intellectual disability. The statistically significant clustering of individuals with mutations in the spectrin domain indicates a recognizable facial phenotype.

a splice variant, c.4860−2A>G. We confirmed pathogenicity by validating the aberrant splicing by PCR and found an extra amplicon in the PCR product of variant c.4860−2A>G (Figure S1). Sanger sequencing results show that the mutation caused disruption to the splice acceptor site and activation of a new splice acceptor site that is 51 base pairs upstream of exon 33; the result was an abnormal intron inclusion transcript (p.Arg1620Serfs*10).

Distinct Neurodevelopmental Phenotypes for Individuals with Either Spectrin or GEFD1 Mutations

Affected individuals of the cohort were born at term with no described antenatal or perinatal complications. All individuals presented with neurodevelopmental delay, but strikingly, the level of intellectual disability was more severe among individuals with a pathogenic variant within the spectrin repeat domain (group 1) than in individuals with a variant in GEFD1 (group 2). Indeed, for group 1, the most frequently observed level of intellectual disability was moderate to severe (22% moderate and 78% severe), whereas individuals within group 2 presented with mild to moderate intellectual disability (50% mild and 25% moderate) (Figure 2A).

Early developmental milestones, including sitting without support, walking, and first words, were delayed in both patient groups, but again, individuals with spectrin mutations were affected more severely (Figure 2B). In

addition, within group 1 there was a greater incidence of individuals' being unable to attain these developmental milestones. For instance, individual 3 (3 years old) was unable to sit unsupported, walk, or talk; individual 4 (4 years old) was unable to walk or talk; and individual 6 (6 years old) was unable to talk (Tables 1 and S1).

Height and weight were reduced in individuals with both spectrin and GEFD1 mutations. The median height for individuals within group 1 was −1.69 SD, while for individuals in group 2 it was −1.34 SD (Figure 2C). In addition, 33% of the group 1 individuals and 14% of group 2 individuals had a height SD that was greater than −2 SD, which is often considered as indicating short stature. Interestingly, weight SD was also reduced to a greater extent in individuals with spectrin variants; there was a mean weight SD of −2.36 in group 1 individuals and a mean weight SD of −2 in group 2 individuals (Figure 2C).

Most strikingly, we noticed a remarkable difference in the occipitofrontal head circumference between individuals with spectrin mutations and those with GEFD1 variants (Figure 2D). Microcephaly, which is defined by a head circumference two SD below the mean, was seen in 100% of group 2 patients, with a mean occipitofrontal circumference (OFC) of −3.82 SD (median OFC of −3.8 SD). In contrast, individuals in group 1 presented with macrocephaly. In the spectrin cohort, 78% had an OFC greater than two SD from the mean; mean OFC was +2.6 SD, median OFC was + 2.7 SD, and the OFC range was between +0.7 SD and +4.7 SD (Figure 2D).

A neurobehavioral phenotype was observed in 19/24 (79%) individuals with a *TRIO* variant, irrespective of the mutated domain. Recurrent behavioral features seen across all individuals included stereotypies (27%), poor attention (70%), obsessive compulsive traits (45%), autistic traits (31%), and aggression (36%) (Figures 2E and 2F). Three individuals (3/18, 17%) were reported to have pain insensitivity or a higher tolerance of pain. There was a diagnosis of epilepsy or evidence of seizure activity in five individuals (24%). Interestingly, seizures were only reported in individuals with mutations in the spectrin domain (3/9) or with truncating mutations (2/5) (Figure S2). Additional symptoms were seen across all mutational domains, including gastrointestinal problems, infantile feeding difficulties, and constipation (Figure S2). Skeletal features, including scoliosis, short tapering fingers, and delayed dental eruption, were also noted in a number of affected individuals. Interestingly, delayed dental eruption was only seen in the individuals with GEFD1 or truncating mutations, and one individual in the spectrin group was reported to have opposing early primary dental eruption. Structural cardiac and brain abnormalities were not observed at a high frequency (Figure S2).

To determine whether individuals had a recognizable facial phenotype, we used quantitative facial phenotyping to analyze photos of the face by using the so-called hybrid model as reported previously[26] (Figures 2G and 2H). 13 individuals had photos, age, ethnicity, and gender available and qualified for analysis. Four of these individuals had mutations in the spectrin domain, and nine had mutations in the GEFD1. No significant difference in CIF was seen between controls and all individuals with TRIO mutations, between controls and individuals with mutations in GEFD1, or between individuals with GEFD1 mutations and those with spectrin mutations. However, persons with mutations in the spectrin domain did cluster significantly when compared to the matched controls (Figures 2G and 2H). This indicates that these individuals have a distinctive facial gestalt.

Taken together, these detailed phenotypic studies of individuals harboring a *TRIO* variant highlight two seemingly distinct clinical syndromes. Individuals with spectrin variants show a more severe developmental phenotype, macrocephaly, and statistically significant clustering of facial dysmorphism, whereas individuals with GEFD1 variants have a less severe developmental phenotype and microcephaly.

The Identified Mutations Target Crucial and Highly Conserved Residues in TRIO

In order to gain insights into the mechanisms by which the mutations identified in the two hotspots could affect TRIO function, we set out to map the position of the mutations on TRIO structure. For the cluster of variants in the seventh spectrin repeat domain, we found that the three residues Thr 1075, Arg 1078, and Asn 1080 are highly conserved across evolution (Figure 3A). Of note, the arginine residue at position 1078 is the most frequently mutated: four unrelated individuals carry a tryptophan, two carry a glutamine, and one carries a glycine residue instead (Table 1). We modeled this seventh spectrin repeat of TRIO on the crystal structures of human β2-spectrin and found that it is composed of three α helices, the second of which carries the cluster of residues 1075 to 1080 (Figure 3B). Axial views of these helices indicate how the changed amino acids might perturb the global arrangement of the helices (Figure 3C). In particular, being larger than the original arginine residue and pointing toward the inside of the domain structure, the mutated Trp 1078 causes an important steric hindrance.

For the GEFD1 cluster of variants, all the mutated amino acids are conserved across evolution and lie within α helices that are also highly conserved among RhoGEFs. These helices (helices α1, α5, and α6) are important for making direct contact with the target GTPase and, hence, for activating the GTPase (Figures 3D and 3E).[43] Given the potentially crucial position of the mutated residues, we asked whether these mutants would be impaired in the binding of the GEFD1 to RAC1. We included the nonsense mutant p.Gln1489Argfs*12 because it also targets a conserved residue in the GEFD1 and thus leads to an incomplete and potentially non-functional GEFD1. To address this question, we performed pulldown assays in HEK293T cells that were co-transfected with a TRIO cDNA carrying the indicated mutations and a $RAC1^{N17}$ construct, which is a

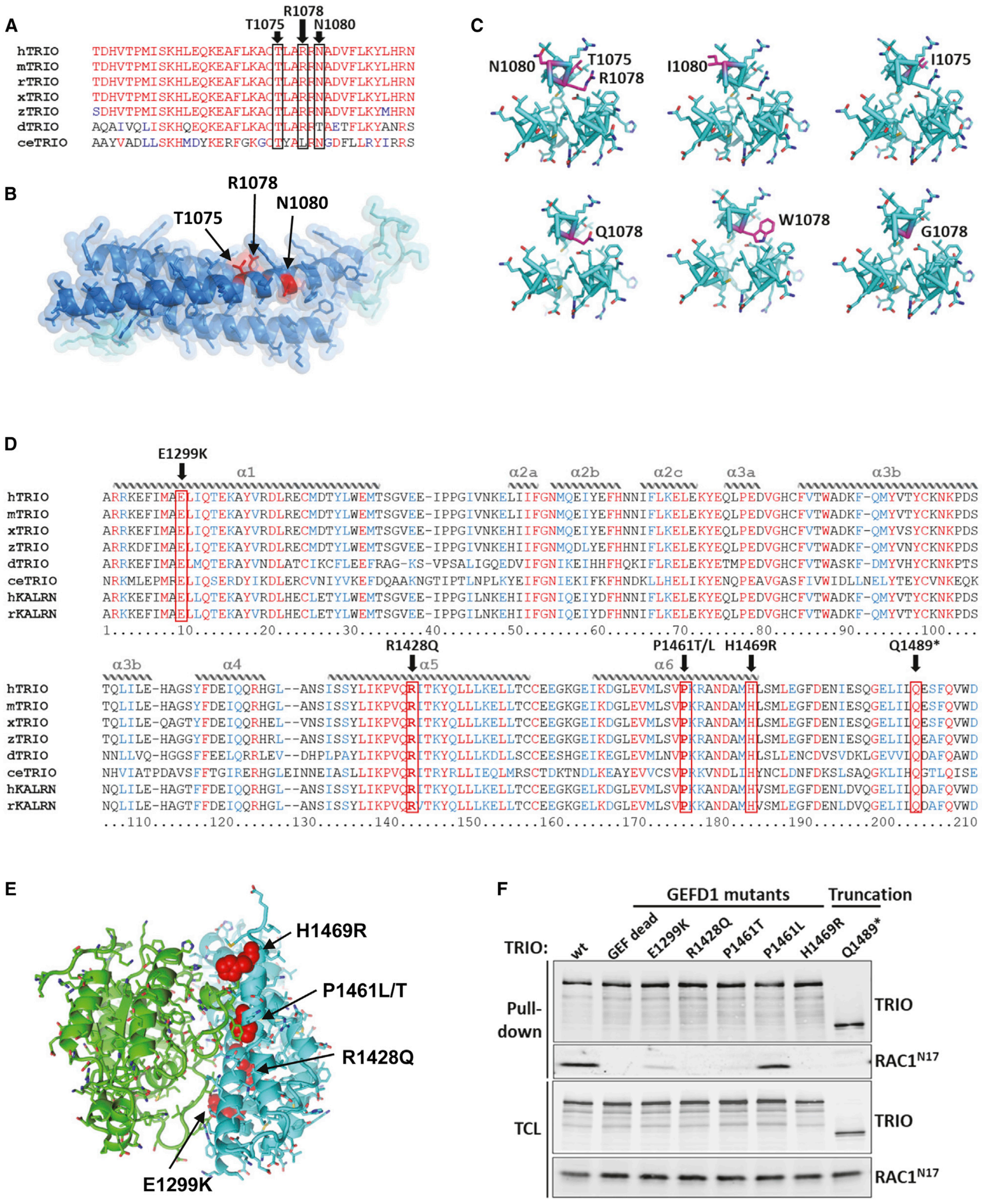

Figure 3. Mapping of the Mutation Sites on the 3D Structure of the TRIO Spectrin 7 Repeat Domain and GEFD1

(A) Species conservation of the residues in TRIO's seventh spectrin repeat, which is mutated in neurodevelopmental diseases. Identical residues are labeled in red, and similar residues are in blue. The positions of the residues p.Thr1075, p.Arg1078, and p.Asn1080 are boxed in black and indicated on top of the sequence, which encompasses amino acids 1053 to 1091, corresponding to the second α helix of the spectrin repeat. Represented species are *Homo sapiens (h), Mus musculus (m), Rattus norvegicus (r), Xenopus laevis (x), Danio rerio (z), Drosophila melanogaster (d),* and *Caenorhabditis elegans (ce).*

(B) Lateral view of the structural model of the seventh spectrin repeat of TRIO. The spectrin domain was modeled based on the crystal structures of human beta2-spectrin (PDB ID 3EDV[30]) with our sequence alignment and SWISS-MODEL server.[32] Parts of the structure

(legend continued on next page)

dominant-negative form of RAC1 that mimics the GDP-bound form of the GTPase and that binds the GEF with higher affinity than WT RAC1 (Figure 3F). Bio-GFP TRIO constructs were purified from the lysates with Streptavidin beads, and the co-purifying $RAC1^{N17}$ was revealed by immunoblotting. As shown in Figure 3F, all the GEFD1 missense mutants and the nonsense p.Gln1489* mutant were unable to bind $RAC1^{N17}$, except that mutant p.Pro1461Leu bound $RAC1^{N17}$ to a similar extent as did WT TRIO. These data confirm the crucial nature of the GEFD1 residues found mutated in individuals with microcephaly.

Spectrin and GEFD1 TRIO Variants Cause Opposite Modulation of RAC1 Activity

We next analyzed whether the mutations identified in individuals affected known functions of TRIO and could thus contribute to the phenotypes observed in these persons.

We tested the effects of the variants on TRIO-mediated activation of the RAC1 signaling pathway. Once in the GTP-bound form, RAC1 is able to bind to the PAK1 kinase and thereby to induce a conformational change that allows for PAK1 auto-phosphorylation. We thus monitored, as a readout for TRIO-mediated RAC1 activation, the levels of PAK1 phosphorylation in HEK293T cells expressing the different TRIO mutants (Figure 4A). All the missense variants in the GEFD1 and the p.Gln1489* nonsense variant led to decreased levels of phosphorylated PAK1 (on Ser 144), except for mutations p.Pro1461Thr and p.Pro1461Leu, which respectively had no effect on or slightly increased phospho-PAK1 levels. In contrast, and surprisingly, all the variants in the seventh spectrin repeat led to a strong increase in PAK1 phosphorylation, most likely reflecting increased RAC1 activation (Figures 4A and 4B). The truncation mutants p.Leu2031* and p.Phe2473* had only a mild and non-significant effect, respectively, on RAC1 activation.

By regulating the remodeling of the actin cytoskeleton, TRIO plays a major role in neurite outgrowth and in the formation of lamellipodia, which are sheet-like actin-rich structures at the leading edge of the cell.[44] To determine whether the GEFD1 and spectrin variants affected these processes, we transfected N1E-115 neuroblastoma cells with the different TRIO mutants and quantified neurite outgrowth (Figures 4C and 4E) and lamellipodia formation both along the shaft and at the tip of the neurite (Figures 4D and 4E). As shown in Figures 4C–4E, the spectrin variants all led to enhanced neurite outgrowth and lamellipodia formation, reflecting almost perfectly the enhanced RAC1 activation induced by these mutants (Figure 4B). In contrast, the GEFD1 and the p.Gln1489* mutants were impaired in forming neurites and lamellipodia, although the p.Pro1461Thr and p.Pro1461Leu mutants were notable exceptions. The truncation mutants Leu2031* and Phe2473* had no effect on these processes.

Truncation in the GEFD1 of TRIO Causes Microcephaly in *X. tropicalis*

Because technical limitations currently prevent us from introducing missense mutations in *X. tropicalis*, we assessed the effect of TRIO truncation variants in this *in vivo* model. The target regions show >97% identity across the surrounding 200 residues between *X. tropicalis* and humans. *X. tropicalis* embryos treated with CRISPR Cas produce targeted indels at such high frequency that the resulting phenotypes can be analyzed in founder embryos.[45–48] In this study, CRISPR Cas targeting the different TRIO domains was injected into fertilized eggs, and its effectiveness at producing indels at the target site was assessed (Figure S3A). The resulting tadpoles were raised to swimming tadpole stages, at which point gross morphology, brain structure, and head diameter were compared and examined. Two single-guide RNAs were tested for each target locus, and each pair produced the same phenotype (A.G. and M.G., unpublished data), strongly suggesting that their effects were target specific. Truncation of GEFD1 (*hs:* Glu 1489, *xt:* Glu 1450) had a clear effect on craniofacial development in that it induced microcephaly in the affected tadpoles, whereas minimal changes were observed in tadpoles with truncation of GEFD2 (*hs*: Leu 2031, *xt*: Leu 1994) (Figures 5A and S3C). Measurement showed that there was no difference in the head diameters of control individuals versus those with the Leu 1994 truncation. The head diameters of individuals with the Gln 1450 truncation were, however,

that were modeled with high and low confidence are in blue and cyan, respectively. Mutations within the spectrin domain seen in affected individuals are indicated in red.

(C) Axial view of the cross section of the structure at the site of the mutations. Mutations are indicated in magenta.

(D) Sequence alignment of the RAC1-specific DH1 domain of TRIO (and KALIRIN) across evolution. Identical residues are labeled in red and similar residues are labeled in blue. The α helices are depicted schematically on top of the sequence alignment. The positions of the alterations p.Glu1299Lys, p.Arg1428Gln, p.Pro1461Leu, p.Pro1461Thr, and p.His1469Arg are indicated in bold and boxed in red. Each mutation affects a highly conserved residue within helices α-1, α-5, and α-6, which make contact with the target GTPase RAC1. Represented species are *Homo sapiens (h), Mus musculus (m), Rattus norvegicus (r), Xenopus laevis (x), Danio rerio (z), Drosophila melanogaster (d),* and *Caenorhabditis elegans (ce)*.

(E) Structure of the DH1 domain of TRIO (cyan) in complex with the small GTPase substrate RAC1 (green). Mutations within the DH1 domain seen in affected individuals are indicated in red and can be seen to occur at the protein-substrate interface. The complex was modeled with the crystal structures of DH1 (PDB: 1NTY) and the complex with substrate (PDB: 1KZ7). Figures of the protein structures were generated with PyMol.[33]

(F) Most of the GEFD1 mutants are affected in their ability to bind to $RAC1^{N17}$ (DN). Immunoblot analysis of a Streptavidin pulldown assay of biotinylated TRIO variants. HEK293T cells were transfected with the indicated biotinylated GFP-TRIO variants and $RAC1^{N17}$. TRIO was pulled down with Streptavidin beads, and the co-precipitating $RAC1^{N17}$ was detected with a RAC1 antibody.

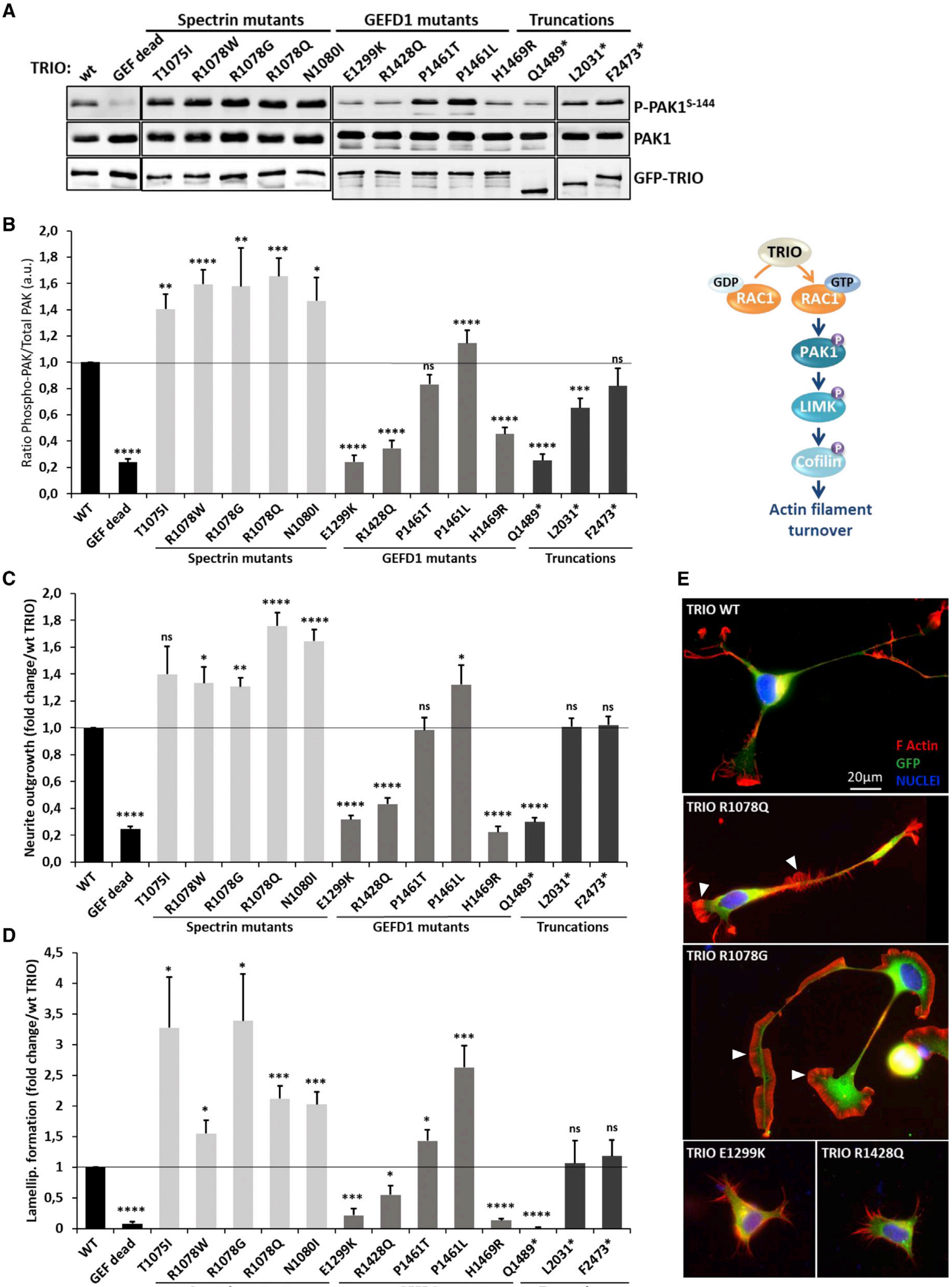

Figure 4. The Spectrin Mutants of TRIO Enhance RAC1 Signaling, Neurite Outgrowth, and Lamellipodia Formation in N1E-115 Cells, Whereas the GEFD1 Mutants Are Mostly Impaired in These Processes

(A) Immunoblot analysis of HEK293T cell lysates transfected with the indicated GFP-TRIO variants and detected with an anti-GFP antibody (lower panel). PAK1 phosphorylation amounts are detected with a phospho-Ser144 PAK1 antibody (upper panel) and compared to total PAK1 amounts detected with a PAK1 antibody (middle panel).

(legend continued on next page)

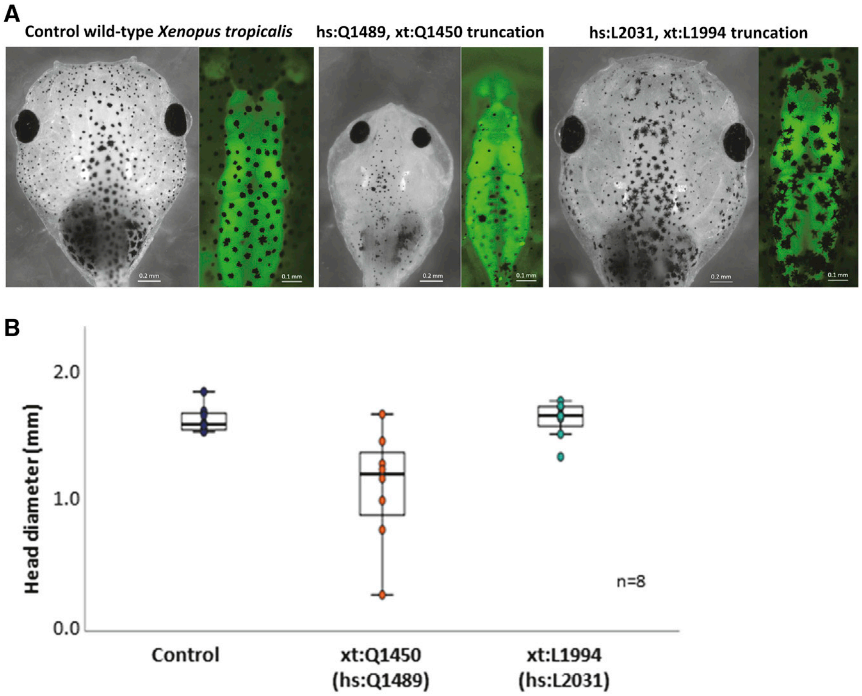

Figure 5. Truncation in the TRIO GEFD1 Reduces Head Size in *X. tropicalis*

(A) *X. tropicalis* eggs from a single female for each of two biological replicates (with parents from different genetic backgrounds) were fertilized with frozen sperm and injected at the one-cell stage with CRISPR-Cas targeting the genome as shown. Representative micrographs for each condition are shown (black and white photos) (all tadpoles are shown in Figure S3). Performing the GEFD1 and GEFD2 frameshift in a tubb2B.GFP *X. tropicalis* showed that forebrain deformities had occurred in 6/8 GEFD1 truncation tadpoles, which was not the case for GEFD2 frameshift. Representative micrographs for each condition are shown (color photos) (all tadpoles are shown in Figure S4).

(B) Comparing the head diameter of the tadpoles carrying truncations in the GEFD1 (hs: Gln 1489* = xt Gln 1450) and GEFD2 (hs: Leu 2031* = xt Leu 1994) domains showed that GEFD1 truncations caused microcephaly, whereas GEFD2 truncations had no effect. Quantification is shown for eight individuals measured (all tadpoles are shown in Figure S3).

smaller than those of both the control group (ANOVA, d.f = 2, F = 9.835, $p = 0.0027153$) and individuals with the Leu 1994 truncation (ANOVA, d.f = 2, F = 9.835, $p = 0.0025286$) (Figures 5B and S3B). This was despite the fact that similar levels of indels were detected in the two groups (Figure S3A). Gross structural abnormalities of the brain were examined within the transgenic line NBT-GFP [Xtr.Tg(tubb2b:GFP) Amaya], which labels differentiated neurons. Embryos with an interruption in GEFD1 showed clear deformation of the forebrain structures, whereas embryos with truncation of GEFD2 (Leu 1994*) were unremarkable (Figures 5A and S4).

Discussion

Here, we report on an important set of individuals harboring pathogenic *TRIO* variants resulting from a variety of nonsense or missense mutations.

We describe various nonsense mutations that are spread along the TRIO sequence. We do not know whether these nonsense mutations generate truncated products of different size or haploinsufficiency after nonsense-mediated decay of the aberrant transcripts. It is likely that all these nonsense mutations will have different functional consequences if they are expressed. For example, the p.Gln1489Argfs*12 variant potentially generates a truncated and, thus, a non-functional GEFD1. Interestingly, the individuals harboring this mutation present with a phenotype similar to that of the patients harboring missense mutations in the GEFD1, suggesting that in this case, the effect of the truncating variant is comparable to the effect of the missense variants in the GEFD1. In contrast, the two truncations p.Leu2031Phefs*9 and p.Phe2473Serfs*54 supposedly produce large proteins with a functional GEFD1, and we show that TRIO-induced RAC1 activation is not affected by these variants. If these truncated variants are expressed, their functional consequences will probably be different from the missense mutations in the GEFD1. Finally, we speculate that the Gln768* variant is probably subject to nonsense-mediated decay, thus generating haploinsufficiency. Characterizing

(B) Quantification of the ratio of phospho-PAK1 amounts over total PAK1 expression. PAK1 phosphorylation is used as a readout for the activation of the RAC1 signaling cascade. Data are presented as the mean ± SEM of at least five independent experiments.
(C) Quantification of the neurite outgrowth induced by WT or mutant TRIO. Neurite outgrowth is monitored on the basis of the number of cells harboring an extension of at least twice the length of the soma. Data are presented as n-fold change over WT TRIO, which was arbitrarily set to 1. Data are presented as the mean ± SEM of at least five independent experiments.
(D) Quantification of lamellipodia formation induced by WT or mutant TRIO. Data are presented as n-fold change over WT TRIO, which was arbitrarily set to 1. Data are presented as the mean ± SEM of at least five independent experiments.
Statistical analysis in (B), (C), and (D) were made by one-way ANOVA followed by Dunnett's test. Asterisks indicate datasets significantly different from WT (*$p < 0.05$, **$p < 0.01$, ***$p < 0.001$, and ****$p < 0.0001$).
(E) Micrographs of N1E-115 cells transfected with the indicated GFP-TRIO variants (green); rhodamine-phalloidin and Hoechst stained the actin (red) and nuclei (blue), respectively. Representative images for each variant type are presented. White arrowheads point to lamellipodia. Scale bar: 20 μm.

the contribution of these variants to the individuals' phenotypes will require further research.

Concerning the missense mutations, we show here that these segregate into two domain-specific hotspots that are associated with differing and distinct clinical phenotypes. We therefore propose that *TRIO*-associated pathologies cover different neurological disorders depending on the mutation location.

Missense mutations that cluster in the GEFD1 of TRIO and affect the activation of RAC1 by TRIO have previously been described.[17–19] Here we describe a second mutational cluster that targets three adjacent residues in the spectrin repeat 7 of TRIO. Compared to the individuals harboring mutations in GEFD1, individuals with missense mutations within the spectrin cluster have more severe intellectual disability, opposing macrocephaly, and statistically significant clustering of facial dysmorphism. It is notable that mutations within the same gene give rise to these strikingly opposite effects on head size, ranging from a median OFC of − 3.8 SD in the GEFD1-mutated individuals to + 2.7 SD in the spectrin-mutated individuals. Interestingly, this important difference in the magnitude of head size has also been described for individuals harboring distinct mutations in *RAC1* (MIM: 602048), the main downstream target of *TRIO*.[49]

To better understand how mutations in *TRIO* could cause such different clinical spectra, we investigated the consequences of these mutations on TRIO activity. The residues mutated in the GEFD1, including the newly identified residues Glu 1299 and His 1469, lie in the α1, α5, and α6 helices and are predicted to be in contact with the Rho GTPases and to thereby catalyze the GDP-to-GTP exchange.[43] Indeed, all the mutants except Pro1461Leu impair RAC1 binding and, consequently, TRIO-mediated RAC1 activation, as measured by PAK1 phosphorylation. In addition, these mutants are impaired in TRIO-mediated neurite outgrowth and lamellipodia formation, which also reflect RAC1 activation.[50] One notable exception is the Pro1461Leu variant, which does not lead to a reduction in RAC1 activation. In contrast, this variant binds RAC1 to a similar extent as WT TRIO and leads to a modest increase in RAC1 activation, as measured by TRIO-induced lamellipodia formation. Interestingly, the two unrelated individuals (individuals 15 and 16) bearing the Pro1461Leu variant are the only two persons who do not present with microcephaly in the GEFD1 cohort. These observations support a correlation between the level of RAC1 activation by TRIO variants and head size. How this variant contributes to the individuals' phenotype will need further investigation.

Interestingly, all the variants we identified in the TRIO spectrin repeat (Thr1075Ile, Arg1078Trp/Gly/Gln, and Asn1080Ile) have a strong TRIO-activating effect, inducing hyperactivation of RAC1. This was measured both by PAK1 phosphorylation and by the formation of actin-rich lamellipodia in N1E-115 cells. Because TRIO-mediated neurite outgrowth is dependent on RAC1 activation, it is not surprising that this process is consistently enhanced by these variants. Contrary to the GEFD1, the spectrin repeats of TRIO are not implicated in the direct activation of RAC1, suggesting that this observed enhanced activity is due to an indirect mechanism. Interestingly, the double TRIO Arg1078Trp/GEFD1-defective mutant is unable to activate RAC1, showing that the hyperactivation of RAC1 by TRIO Arg1078Trp is strictly dependent on the activity of GEFD1 (M.B. and D.L., unpublished data). Based on these data, we propose that mutations in the spectrin cluster perturb mutual arrangement of α helices in the seventh spectrin repeat and affect overall TRIO fold and/or signaling, leading to hyperactivation of RAC1 by TRIO. The spectrin domains within TRIO are known to bind different TRIO regulators, such as Kidins220, DISC1, and NAV1.[34,51,52] It has also been proposed that spectrin repeats inhibit TRIO-mediated RAC1 activation by intramolecular binding to the GEFD1 and thus prevent TRIO-RAC1 binding and that the binding of DISC1 releases this intramolecular inhibition.[52] Whether the mutations found in affected individuals perturb this intramolecular binding or the binding to different partners will be the subject of further investigation.

This hypothesis is also supported by our molecular modeling studies based on the crystal structure of the human β2 spectrin. We observed that the cluster of mutations identified in affected individuals lies in the second α helix of the spectrin repeat. The Arg 1078 residue faces another α helix, whereas the Thr 1075 and Asn 1080 residues are exposed on the external side of the helix. Interestingly, individuals harboring TRIO Arg 1078 variants display the most severe phenotype. In addition, among the TRIO Arg 1078 variants, Arg1078Trp is the most frequent and gives rise to the strongest phenotype. The tryptophan brings an important steric constraint as a result of its large size, suggesting that mutations of the Arg 1078 residue probably affect the structural organization of the three α helices of spectrin repeat 7, leading to perturbed TRIO folding and/or binding to its partners and ultimately altering TRIO activity.

Combined, our data show that mutations in two different hotspots negatively or positively affect RAC1 activation by TRIO and indicate that these mutations represent loss- or gain-of-function mutations that give rise to distinct developmental defects. Our data show that the different RAC1 activation levels dependent on the mutation are well correlated to the head size of the affected individuals. In line with this, *TRIO* depletion in mice causes neurodevelopmental deficits associated with a decrease in brain size.[13,14] This was also supported by our experiments in *Xenopus* expressing different TRIO variants that resulted in phenotypes concordant with those of affected humans. This finding adds to the evidence that F0 *X. tropicalis* embryos produced by gene editing can be used effectively in the study of human gene variants. Indeed, mimicking a GEFD1 variant in *Xenopus* produces microcephaly as in humans, whereas making a variant with a truncated

GEFD2 has little effect in either humans or frogs. Unfortunately, technical limitations currently prevent us from introducing missense mutations in *X. tropicalis*, and therefore, we could not directly test whether a missense mutation overactivating RAC1 in group 1 would induce macrocephaly in the frog. Nonetheless, our data provide strong evidence that missense *TRIO* mutations inducing RAC1 overactivation are associated with macrocephaly. Indeed, in our study we describe seven independent individuals who have the same targeted residue (Arg1078Trp/Gly/Gln) and all present with macrocephaly. This important number of independent individuals with a similar phenotype reduces the potential contribution of other variants to the phenotype and strongly suggests that hyperactivation of RAC1 by TRIO is associated with macrocephaly.

Perturbation of head size often results from dysregulation of early developmental pathways, such as proliferation and/or apoptosis or neural migration of the cortex, in neural progenitor cells (NPCs). Interestingly, *RAC1* deficiency in the forebrain of mice leads to a smaller forebrain resembling the microcephaly phenotype.[53] It has been proposed that this microcephaly results from a reduction of NPCs as a result of an accelerated cell-cycle exit, combined with increased apoptosis of nascent neurons and disrupted differentiation of post-mitotic neurons.[54] Of note, we have previously shown that TRIO is important for cytokinesis,[7] suggesting that perturbation of TRIO activity could affect the cell cycle of NPCs.

Given the large body of evidence implicating TRIO in different aspects of brain development, we speculate that *TRIO* mutations might affect other neuronal processes, including axon guidance and the formation of synapses. This hypothesis is reinforced by previous data showing that conditional deletion of *RAC1* and *RAC3* in mouse neurons leads to migration, differentiation, and connectivity defects.[55–57] Perturbation of synapses by *TRIO* depletion or by missense mutations has already been described *in vitro* and *in vivo*.[10,16,18] TRIO is an important component of the netrin-1-dependent pathway, which is important for axon guidance, and *TRIO* depletion in mice results in defects in the corpus callosum's axonal projections, which are netrin-1 dependent.[58] Interestingly, we describe here numerous structural brain anomalies in two affected individuals, one of whom has partial corpus callosum agenesis with an interhemispheric cyst and the other of whom has loss of white matter volume, asymmetric enlargement of the ventricles, and a thin corpus callosum. These data suggest that *TRIO*-affected individuals might also exhibit defects in the axonal guidance process during early development.

Our data also highlight the importance of strict control of the level of RAC1 activation for proper neuronal development. In conjunction with this, distinct *RAC1* variants have been shown to have a positive or negative effect on RAC1 function and to cause diverse phenotypes.[49] Distinct phenotypes that correlate to activating or inactivating mutations at different locations in the gene have also been described in *PAK1* and *PAK3* (MIM: 618158).[59–61] Together, our data reinforce the hypothesis that the TRIO/RAC1/PAK axis is a major pathway for neuronal development that is perturbed in neurodevelopmental diseases. Interestingly, PAK1 inhibition has been used to reverse behavior defects in mice.[59,62] These observations suggest an avenue for therapeutic intervention for *TRIO* disorders associated with a hyperactivation of RAC1 via a pharmacological strategy targeting PAK1 activation.

In conclusion, we have identified two clusters of *TRIO* mutations associated with distinct clinical and neurodevelopmental phenotypes attributable to differing levels of RAC1 activation. We suggest a genotype-phenotype correlation based on the location of the mutation; this correlation will serve as a useful adjunct for clinical evaluation and the interpretation of variant pathogenicity. In addition, and by using the previously reported hybrid model for quantitative facial phenotyping, we see that the spectrin-mutated group has a distinctive facial gestalt assessable through the algorithms.

Supplemental Data

Supplemental Data can be found online at https://doi.org/10.1016/j.ajhg.2020.01.018.

Acknowledgments

We are grateful to all the patients and their families for their participation in this study. We thank all members of the Debant team for helpful discussions. We acknowledge the imaging facility Montpellier Ressources Imagerie (MRI), a member of the national France-BioImaging infrastructure supported by the French National Research Agency (ANR-10-INBS-04, "Investments for the future"). We thank A. Guille for help with statistical analysis. This work was supported by grants from the Fondation pour la Recherche Médicale (Equipes FRM 2016, DEQ20160334942) to A.D. A PhD fellowship from the Ministère de l'Enseignement Supérieur et de la Recherche supported M.B. The European Xenopus Resource Centre (EXRC) is funded by the Wellcome Trust (212942/Z/18/Z) and the Biotechnology and Biological Sciences Research Council (BB/R014841/1). The Baralle lab is supported by a National Institute for Health Research research professorship to D.B. (RP-2016-07-011). S.G.-H. is funded by a research fellowship from the Health Education England Genomics Education Programme. The DDD study presents independent research commissioned by the Health Innovation Challenge Fund [grant HICF-1009-003]. This research was made possible through access to data and findings generated by the 100,000 Genomes Project, which is managed by Genomics England Limited (a wholly owned company of the Department of Health and Social Care). The 100,000 Genomes Project is funded by the National Institute for Health Research and NHS England. The Wellcome Trust, Cancer Research UK, and the Medical Research Council have also funded research infrastructure. The 100,000 Genomes Project uses data provided by patients and collected by the National Health Service as part of their care and support.

Declaration of Interests

The authors declare no competing interests.

Received: October 11, 2019
Accepted: January 27, 2020
Published: February 27, 2020

Web Resources

CRISPRscan, https://www.crisprscan.org/
DDD UK, https://www.ddduk.org/access.html
DECIPHER, https://decipher.sanger.ac.uk/
The National Genomics Research and Healthcare Knowledgebase v5, https://www.genomicsengland.co.uk/the-national-genomics-research-and-healthcare-knowledgebase/
Online Mendelian Inheritance in Man (OMIM), https://www.omim.org/
Primer3, http://primer3.ut.ee
Xenbase, http://www.xenbase.org/entry; V9.1 of the genome

References

1. Govek, E.E., Newey, S.E., and Van Aelst, L. (2005). The role of the Rho GTPases in neuronal development. Genes Dev. *19*, 1–49.
2. Tolias, K.F., Duman, J.G., and Um, K. (2011). Control of synapse development and plasticity by Rho GTPase regulatory proteins. Prog. Neurobiol. *94*, 133–148.
3. Debant, A., Serra-Pagès, C., Seipel, K., O'Brien, S., Tang, M., Park, S.H., and Streuli, M. (1996). The multidomain protein Trio binds the LAR transmembrane tyrosine phosphatase, contains a protein kinase domain, and has separate rac-specific and rho-specific guanine nucleotide exchange factor domains. Proc. Natl. Acad. Sci. USA *93*, 5466–5471.
4. Blangy, A., Vignal, E., Schmidt, S., Debant, A., Gauthier-Rouvière, C., and Fort, P. (2000). TrioGEF1 controls Rac- and Cdc42-dependent cell structures through the direct activation of rhoG. J. Cell Sci. *113*, 729–739.
5. Bellanger, J.M., Lazaro, J.B., Diriong, S., Fernandez, A., Lamb, N., and Debant, A. (1998). The two guanine nucleotide exchange factor domains of Trio link the Rac1 and the RhoA pathways in vivo. Oncogene *16*, 147–152.
6. Schmidt, S., and Debant, A. (2014). Function and regulation of the Rho guanine nucleotide exchange factor Trio. Small GTPases *5*, e29769.
7. Cannet, A., Schmidt, S., Delaval, B., and Debant, A. (2014). Identification of a mitotic Rac-GEF, Trio, that counteracts MgcRacGAP function during cytokinesis. Mol. Biol. Cell *25*, 4063–4071.
8. Iyer, S.C., Wang, D., Iyer, E.P., Trunnell, S.A., Meduri, R., Shinwari, R., Sulkowski, M.J., and Cox, D.N. (2012). The RhoGEF trio functions in sculpting class specific dendrite morphogenesis in Drosophila sensory neurons. PLoS ONE *7*, e33634.
9. Ba, W., van der Raadt, J., and Nadif Kasri, N. (2013). Rho GTPase signaling at the synapse: implications for intellectual disability. Exp. Cell Res. *319*, 2368–2374.
10. Herring, B.E., and Nicoll, R.A. (2016). Kalirin and Trio proteins serve critical roles in excitatory synaptic transmission and LTP. Proc. Natl. Acad. Sci. USA *113*, 2264–2269.
11. O'Brien, S.P., Seipel, K., Medley, Q.G., Bronson, R., Segal, R., and Streuli, M. (2000). Skeletal muscle deformity and neuronal disorder in Trio exchange factor-deficient mouse embryos. Proc. Natl. Acad. Sci. USA *97*, 12074–12078.
12. Peng, Y.J., He, W.Q., Tang, J., Tao, T., Chen, C., Gao, Y.Q., Zhang, W.C., He, X.Y., Dai, Y.Y., Zhu, N.C., et al. (2010). Trio is a key guanine nucleotide exchange factor coordinating regulation of the migration and morphogenesis of granule cells in the developing cerebellum. J. Biol. Chem. *285*, 24834–24844.
13. Zong, W., Liu, S., Wang, X., Zhang, J., Zhang, T., Liu, Z., Wang, D., Zhang, A., Zhu, M., and Gao, J. (2015). Trio gene is required for mouse learning ability. Brain Res. *1608*, 82–90.
14. Katrancha, S.M., Shaw, J.E., Zhao, A.Y., Myers, S.A., Cocco, A.R., Jeng, A.T., Zhu, M., Pittenger, C., Greer, C.A., Carr, S.A., et al. (2019). Trio Haploinsufficiency Causes Neurodevelopmental Disease-Associated Deficits. Cell Rep. *26*, 2805–2817.e9.
15. Tao, T., Sun, J., Peng, Y., Wang, P., Chen, X., Zhao, W., Li, Y., Wei, L., Wang, W., Zheng, Y., et al. (2019). Distinct functions of Trio GEF domains in axon outgrowth of cerebellar granule neurons. J. Genet. Genomics *46*, 87–96.
16. Ba, W., Yan, Y., Reijnders, M.R., Schuurs-Hoeijmakers, J.H., Feenstra, I., Bongers, E.M., Bosch, D.G., De Leeuw, N., Pfundt, R., Gilissen, C., et al. (2016). TRIO loss of function is associated with mild intellectual disability and affects dendritic branching and synapse function. Hum. Mol. Genet. *25*, 892–902.
17. Pengelly, R.J., Greville-Heygate, S., Schmidt, S., Seaby, E.G., Jabalameli, M.R., Mehta, S.G., Parker, M.J., Goudie, D., Fagotto-Kaufmann, C., Mercer, C., et al.; DDD Study (2016). Mutations specific to the Rac-GEF domain of *TRIO* cause intellectual disability and microcephaly. J. Med. Genet. *53*, 735–742.
18. Sadybekov, A., Tian, C., Arnesano, C., Katritch, V., and Herring, B.E. (2017). An autism spectrum disorder-related de novo mutation hotspot discovered in the GEF1 domain of Trio. Nat. Commun. *8*, 601.
19. Katrancha, S.M., Wu, Y., Zhu, M., Eipper, B.A., Koleske, A.J., and Mains, R.E. (2017). Neurodevelopmental disease-associated de novo mutations and rare sequence variants affect TRIO GDP/GTP exchange factor activity. Hum. Mol. Genet. *26*, 4728–4740.
20. Petrovski, S., Wang, Q., Heinzen, E.L., Allen, A.S., and Goldstein, D.B. (2013). Genic intolerance to functional variation and the interpretation of personal genomes. PLoS Genet. *9*, e1003709.
21. Deciphering Developmental Disorders Study (2015). Large-scale discovery of novel genetic causes of developmental disorders. Nature *519*, 223–228.
22. McLaren, W., Gil, L., Hunt, S.E., Riat, H.S., Ritchie, G.R., Thormann, A., Flicek, P., and Cunningham, F. (2016). The Ensembl Variant Effect Predictor. Genome Biol. *17*, 122.
23. Desmet, F.-O., Hamroun, D., Lalande, M., Collod-Béroud, G., Claustres, M., and Béroud, C. (2009). Human Splicing Finder: an online bioinformatics tool to predict splicing signals. Nucleic Acids Res. *37*, e67.
24. Yeo, G., and Burge, C.B. (2004). Maximum entropy modeling of short sequence motifs with applications to RNA splicing signals. J. Comput. Biol. *11*, 377–394.
25. Reese, M.G., Eeckman, F.H., Kulp, D., and Haussler, D. (1997). Improved splice site detection in Genie. J. Comput. Biol. *4*, 311–323.
26. van der Donk, R., Jansen, S., Schuurs-Hoeijmakers, J.H.M., Koolen, D.A., Goltstein, L.C.M.J., Hoischen, A., Brunner, H.G., Kemmeren, P., Nellåker, C., Vissers, L.E.L.M., et al. (2019). Next-generation phenotyping using computer vision

algorithms in rare genomic neurodevelopmental disorders. Genet. Med. *21*, 1719–1725.
27. Diets, I.J., van der Donk, R., Baltrunaite, K., Waanders, E., Reijnders, M.R.F., Dingemans, A.J.M., Pfundt, R., Vulto-van Silfhout, A.T., Wiel, L., Gilissen, C., et al. (2019). De Novo and Inherited Pathogenic Variants in KDM3B Cause Intellectual Disability, Short Stature, and Facial Dysmorphism. Am. J. Hum. Genet. *104*, 758–766.
28. Amos, B., Ludwiczuk, B., and Satyanarayanan, M. (2016). OpenFace: A general-purpose face recognition library with mobile applications. (Carnegie Mellon School of Computer Science), CMU–CD–16–188.
29. Ferry, Q., Steinberg, J., Webber, C., FitzPatrick, D.R., Ponting, C.P., Zisserman, A., and Nellåker, C. (2014). Diagnostically relevant facial gestalt information from ordinary photos. eLife *3*, e02020.
30. Davis, L., Abdi, K., Machius, M., Brautigam, C., Tomchick, D.R., Bennett, V., and Michaely, P. (2009). Localization and structure of the ankyrin-binding site on beta2-spectrin. J. Biol. Chem. *284*, 6982–6987.
31. Bucher, P., Karplus, K., Moeri, N., and Hofmann, K. (1996). A flexible motif search technique based on generalized profiles. Comput. Chem. *20*, 3–23.
32. Waterhouse, A., Bertoni, M., Bienert, S., Studer, G., Tauriello, G., Gumienny, R., Heer, F.T., de Beer, T.A.P., Rempfer, C., Bordoli, L., et al. (2018). SWISS-MODEL: homology modelling of protein structures and complexes. Nucleic Acids Res. *46* (W1), W296–W303.
33. DeLano, W.L. (2002). The PyMOL Molecular Graphics System (San Carlos: Delano Scientific).
34. van Haren, J., Boudeau, J., Schmidt, S., Basu, S., Liu, Z., Lammers, D., Demmers, J., Benhari, J., Grosveld, F., Debant, A., and Galjart, N. (2014). Dynamic microtubules catalyze formation of navigator-TRIO complexes to regulate neurite extension. Curr. Biol. *24*, 1778–1785.
35. Estrach, S., Schmidt, S., Diriong, S., Penna, A., Blangy, A., Fort, P., and Debant, A. (2002). The Human Rho-GEF trio and its target GTPase RhoG are involved in the NGF pathway, leading to neurite outgrowth. Curr. Biol. *12*, 307–312.
36. DeGeer, J., Boudeau, J., Schmidt, S., Bedford, F., Lamarche-Vane, N., and Debant, A. (2013). Tyrosine phosphorylation of the Rho guanine nucleotide exchange factor Trio regulates netrin-1/DCC-mediated cortical axon outgrowth. Mol. Cell. Biol. *33*, 739–751.
37. Sargent, M.G., and Mohun, T.J. (2005). Cryopreservation of sperm of Xenopus laevis and Xenopus tropicalis. Genesis *41*, 41–46.
38. Mansour, N., Lahnsteiner, F., and Patzner, R.A. (2009). Optimization of the cryopreservation of African clawed frog (Xenopus laevis) sperm. Theriogenology *72*, 1221–1228.
39. Pearl, E., Morrow, S., Noble, A., Lerebours, A., Horb, M., and Guille, M. (2017). An optimized method for cryogenic storage of Xenopus sperm to maximise the effectiveness of research using genetically altered frogs. Theriogenology *92*, 149–155.
40. Nakayama, T., Blitz, I.L., Fish, M.B., Odeleye, A.O., Manohar, S., Cho, K.W., and Grainger, R.M. (2014). Cas9-based genome editing in Xenopus tropicalis. Methods Enzymol. *546*, 355–375.
41. Guille, M. (1999). Molecular Methods in Developmental Biology: Xenopus and Zebrafish, *Volume 27* (Springer Science & Business Media).
42. Vouillot, L., Thélie, A., and Pollet, N. (2015). Comparison of T7E1 and surveyor mismatch cleavage assays to detect mutations triggered by engineered nucleases. G3 (Bethesda) *5*, 407–415.
43. Chhatriwala, M.K., Betts, L., Worthylake, D.K., and Sondek, J. (2007). The DH and PH domains of Trio coordinately engage Rho GTPases for their efficient activation. J. Mol. Biol. *368*, 1307–1320.
44. Ladwein, M., and Rottner, K. (2008). On the Rho'd: the regulation of membrane protrusions by Rho-GTPases. FEBS Lett. *582*, 2066–2074.
45. Deniz, E., Mis, E.K., Lane, M., and Khokha, M.K. (2018). CRISPR/Cas9 F0 Screening of Congenital Heart Disease Genes in Xenopus tropicalis. Methods Mol. Biol. *1865*, 163–174.
46. Naert, T., Colpaert, R., Van Nieuwenhuysen, T., Dimitrakopoulou, D., Leoen, J., Haustraete, J., Boel, A., Steyaert, W., Lepez, T., Deforce, D., et al. (2016). CRISPR/Cas9 mediated knockout of rb1 and rbl1 leads to rapid and penetrant retinoblastoma development in Xenopus tropicalis. Sci. Rep. *6*, 35264.
47. Naert, T., and Vleminckx, K. (2018). CRISPR/Cas9 disease models in zebrafish and Xenopus: The genetic renaissance of fish and frogs. Drug Discov. Today. Technol. *28*, 41–52.
48. Sega, A.G., Mis, E.K., Lindstrom, K., Mercimek-Andrews, S., Ji, W., Cho, M.T., Juusola, J., Konstantino, M., Jeffries, L., Khokha, M.K., and Lakhani, S.A. (2019). De novo pathogenic variants in neuronal differentiation factor 2 (NEUROD2) cause a form of early infantile epileptic encephalopathy. J. Med. Genet. *56*, 113–122.
49. Reijnders, M.R.F., Ansor, N.M., Kousi, M., Yue, W.W., Tan, P.L., Clarkson, K., Clayton-Smith, J., Corning, K., Jones, J.R., Lam, W.W.K., et al.; Deciphering Developmental Disorders Study (2017). RAC1 Missense Mutations in Developmental Disorders with Diverse Phenotypes. Am. J. Hum. Genet. *101*, 466–477.
50. Meyer, G., and Feldman, E.L. (2002). Signaling mechanisms that regulate actin-based motility processes in the nervous system. J. Neurochem. *83*, 490–503.
51. Neubrand, V.E., Thomas, C., Schmidt, S., Debant, A., and Schiavo, G. (2010). Kidins220/ARMS regulates Rac1-dependent neurite outgrowth by direct interaction with the RhoGEF Trio. J. Cell Sci. *123*, 2111–2123.
52. Chen, S.Y., Huang, P.H., and Cheng, H.J. (2011). Disrupted-in-Schizophrenia 1-mediated axon guidance involves TRIO-RAC-PAK small GTPase pathway signaling. Proc. Natl. Acad. Sci. USA *108*, 5861–5866.
53. Chen, L., Melendez, J., Campbell, K., Kuan, C.-Y., and Zheng, Y. (2009). Rac1 deficiency in the forebrain results in neural progenitor reduction and microcephaly. Dev. Biol. *325*, 162–170.
54. Leone, D.P., Srinivasan, K., Brakebusch, C., and McConnell, S.K. (2010). The rho GTPase Rac1 is required for proliferation and survival of progenitors in the developing forebrain. Dev. Neurobiol. *70*, 659–678.
55. Vaghi, V., Pennucci, R., Talpo, F., Corbetta, S., Montinaro, V., Barone, C., Croci, L., Spaiardi, P., Consalez, G.G., Biella, G., and de Curtis, I. (2014). Rac1 and rac3 GTPases control synergistically the development of cortical and hippocampal GABAergic interneurons. Cereb. Cortex *24*, 1247–1258.
56. Corbetta, S., Gualdoni, S., Ciceri, G., Monari, M., Zuccaro, E., Tybulewicz, V.L., and de Curtis, I. (2009). Essential role of Rac1

and Rac3 GTPases in neuronal development. FASEB J. *23*, 1347–1357.

57. Pennucci, R., Tavano, S., Tonoli, D., Gualdoni, S., and de Curtis, I. (2011). Rac1 and Rac3 GTPases regulate the development of hilar mossy cells by affecting the migration of their precursors to the hilus. PLoS ONE *6*, e24819.

58. Briançon-Marjollet, A., Ghogha, A., Nawabi, H., Triki, I., Auziol, C., Fromont, S., Piché, C., Enslen, H., Chebli, K., Cloutier, J.F., et al. (2008). Trio mediates netrin-1-induced Rac1 activation in axon outgrowth and guidance. Mol. Cell. Biol. *28*, 2314–2323.

59. Harms, F.L., Kloth, K., Bley, A., Denecke, J., Santer, R., Lessel, D., Hempel, M., and Kutsche, K. (2018). Activating Mutations in PAK1, Encoding p21-Activated Kinase 1, Cause a Neurodevelopmental Disorder. Am. J. Hum. Genet. *103*, 579–591.

60. Allen, K.M., Gleeson, J.G., Bagrodia, S., Partington, M.W., MacMillan, J.C., Cerione, R.A., Mulley, J.C., and Walsh, C.A. (1998). PAK3 mutation in nonsyndromic X-linked mental retardation. Nat. Genet. *20*, 25–30.

61. Horn, S., Au, M., Basel-Salmon, L., Bayrak-Toydemir, P., Chapin, A., Cohen, L., Elting, M.W., Graham, J.M., Gonzaga-Jauregui, C., Konen, O., et al. (2019). De novo variants in PAK1 lead to intellectual disability with macrocephaly and seizures. Brain *142*, 3351–3359. https://doi.org/10.1093/brain/awz264.

62. Dolan, B.M., Duron, S.G., Campbell, D.A., Vollrath, B., Shankaranarayana Rao, B.S., Ko, H.Y., Lin, G.G., Govindarajan, A., Choi, S.Y., and Tonegawa, S. (2013). Rescue of fragile X syndrome phenotypes in Fmr1 KO mice by the small-molecule PAK inhibitor FRAX486. Proc. Natl. Acad. Sci. USA *110*, 5671–5676.

The American Journal of Human Genetics, Volume *106*

Supplemental Data

Opposite Modulation of RAC1 by Mutations in *TRIO* Is Associated with Distinct, Domain-Specific Neurodevelopmental Disorders

Sónia Barbosa, Stephanie Greville-Heygate, Maxime Bonnet, Annie Godwin, Christine Fagotto-Kaufmann, Andrey V. Kajava, Damien Laouteouet, Rebecca Mawby, Htoo Aung Wai, Alexander J.M. Dingemans, Jayne Hehir-Kwa, Marjorlaine Willems, Yline Capri, Sarju G. Mehta, Helen Cox, David Goudie, Fleur Vansenne, Peter Turnpenny, Marie Vincent, Benjamin Cogné, Gaëtan Lesca, Jozef Hertecant, Diana Rodriguez, Boris Keren, Lydie Burglen, Marion Gérard, Audrey Putoux, C4RCD Research Group, Vincent Cantagrel, Karine Siquier-Pernet, Marlene Rio, Siddharth Banka, Ajoy Sarkar, Marcie Steeves, Michael Parker, Emma Clement, Sébastien Moutton, Frédéric Tran Mau-Them, Amélie Piton, Bert B.A. de Vries, Matthew Guille, Anne Debant, Susanne Schmidt, and Diana Baralle

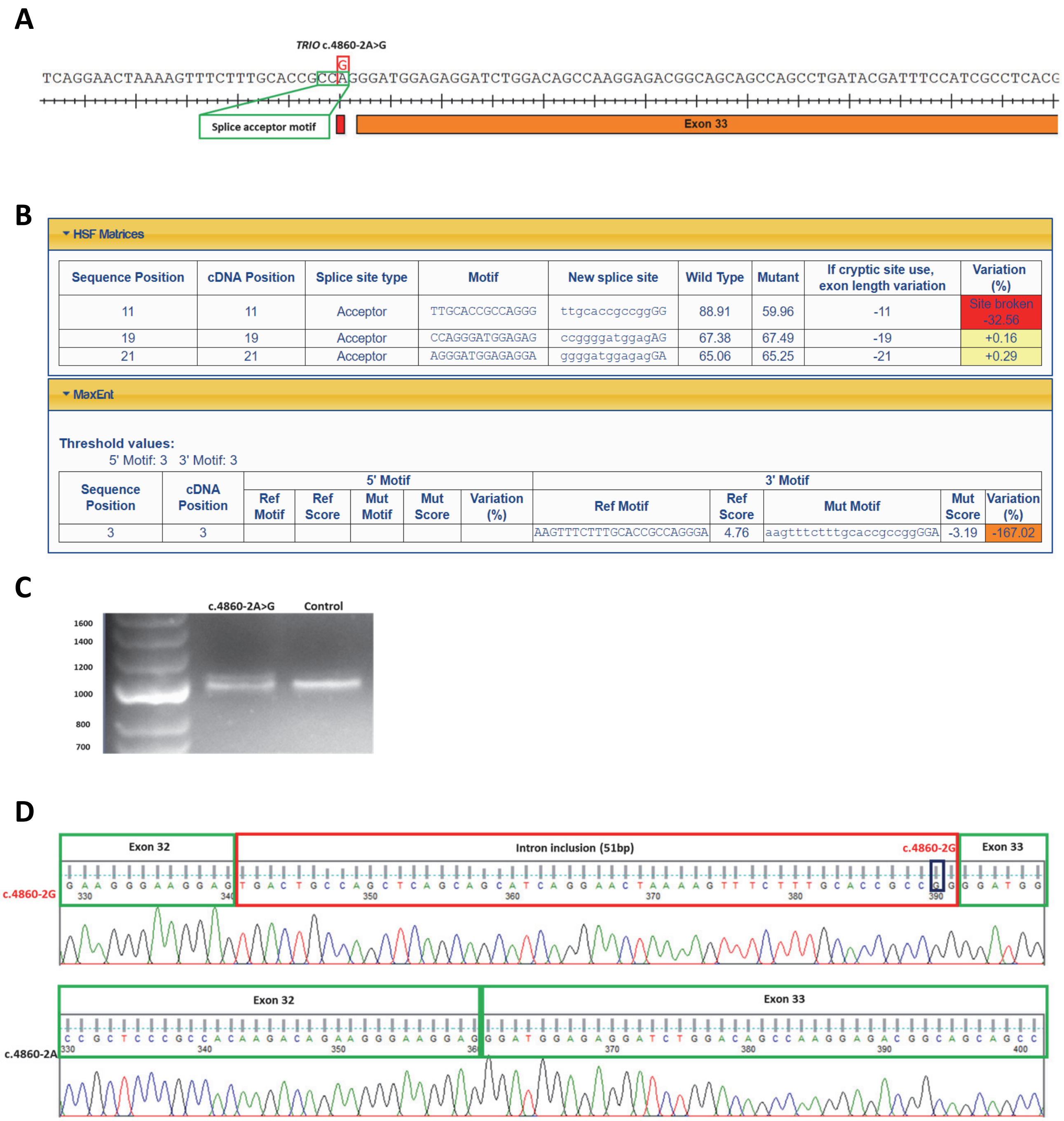

HSF Matrices

Sequence Position	cDNA Position	Splice site type	Motif	New splice site	Wild Type	Mutant	If cryptic site use, exon length variation	Variation (%)
11	11	Acceptor	TTGCACCGCCAGGG	ttgcaccgccggGG	88.91	59.96	-11	Site broken -32.56
19	19	Acceptor	CCAGGGATGGAGAG	ccggggatggagAG	67.38	67.49	-19	+0.16
21	21	Acceptor	AGGGATGGAGAGGA	ggggatggagagGA	65.06	65.25	-21	+0.29

MaxEnt

Threshold values:
5' Motif: 3 3' Motif: 3

Sequence Position	cDNA Position	5' Motif					3' Motif				
		Ref Motif	Ref Score	Mut Motif	Mut Score	Variation (%)	Ref Motif	Ref Score	Mut Motif	Mut Score	Variation (%)
3	3						AAGTTTCTTTGCACCGCCAGGGA	4.76	aagtttctttgcaccgccggGGA	-3.19	-167.02

Figure S1: Impact of splice acceptor site variant in *TRIO* (c.4860-2A>G) on aberrant splicing. A. c.4860-2A>G is located in splice acceptor motif site of Exon 33. **B.** Human Splice Site Finder (HSF Version 3.1) and MaxEntScan predicted splice acceptor site broken. **C.** Gel electrophoresis shows an extra band in c.4860-2A>G PCR. **D.** Sanger sequencing shows an extra 51 nucleotides between Exon 32 and Exon 33.

Figure S1

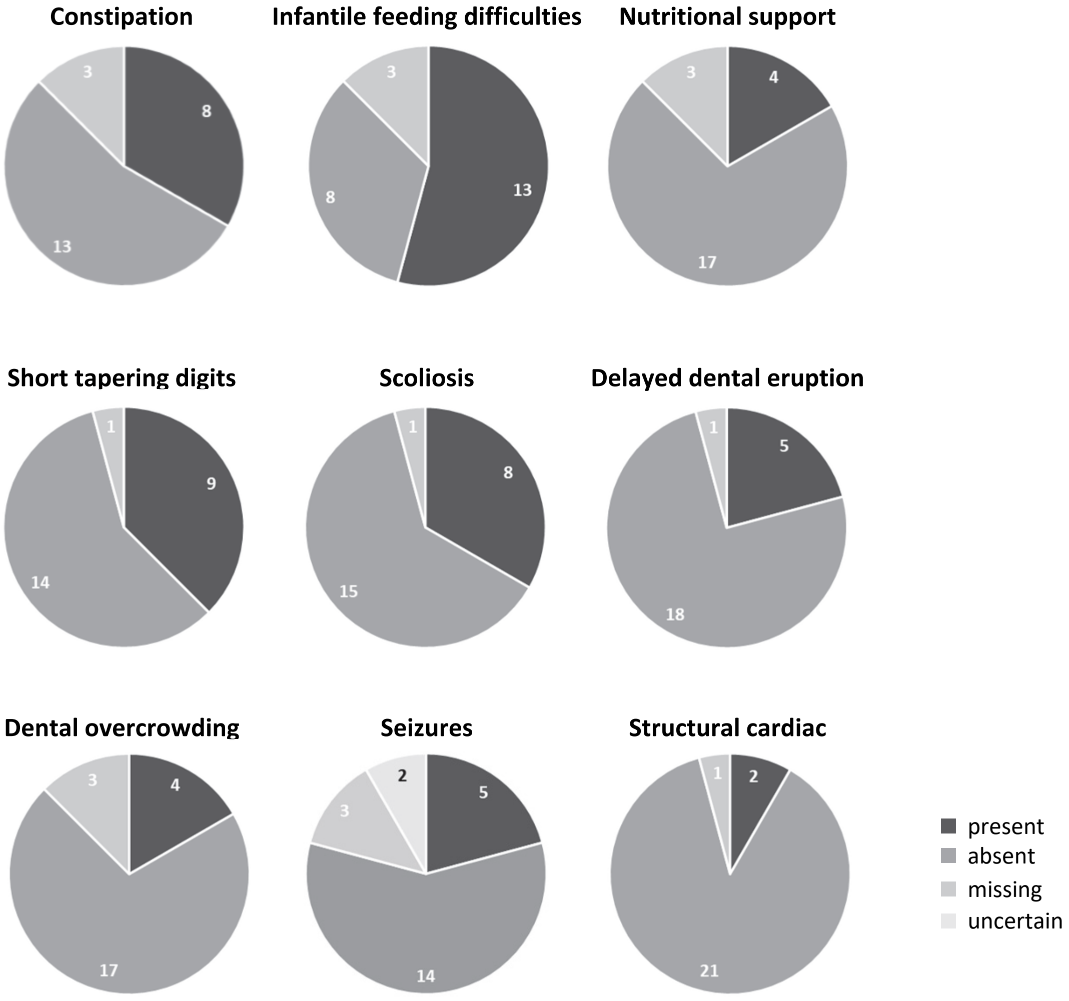

Figure S2: Expanded phenotype characteristics across the cohort. Gastrointestinal features including infantile feeding difficulties and constipation where frequently observed. Skeletal manifestations included short tapering digits, scoliosis and delayed dental eruption were also noted. Classified as: present, dark grey; absent, middle grey; missing, light grey and uncertain, very light grey.

A

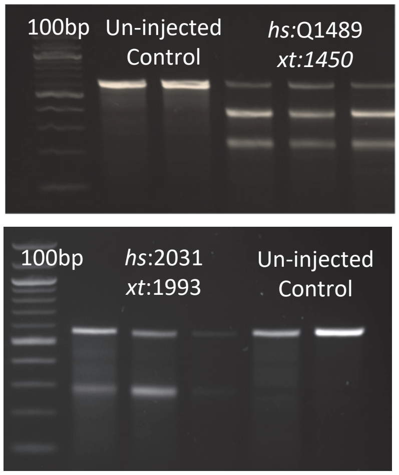

B

Head diameter (mm)			
	Control	Q1450	L1994
1	1.712	1.018	1.533
2	1.619	1.189	1.674
3	1.553	1.302	1.753
4	1.581	1.258	1.743
5	1.864	0.79	1.793
6	1.551	1.479	1.357
7	1.6	0.283	1.683
8	1.68	1.688	1.657

C

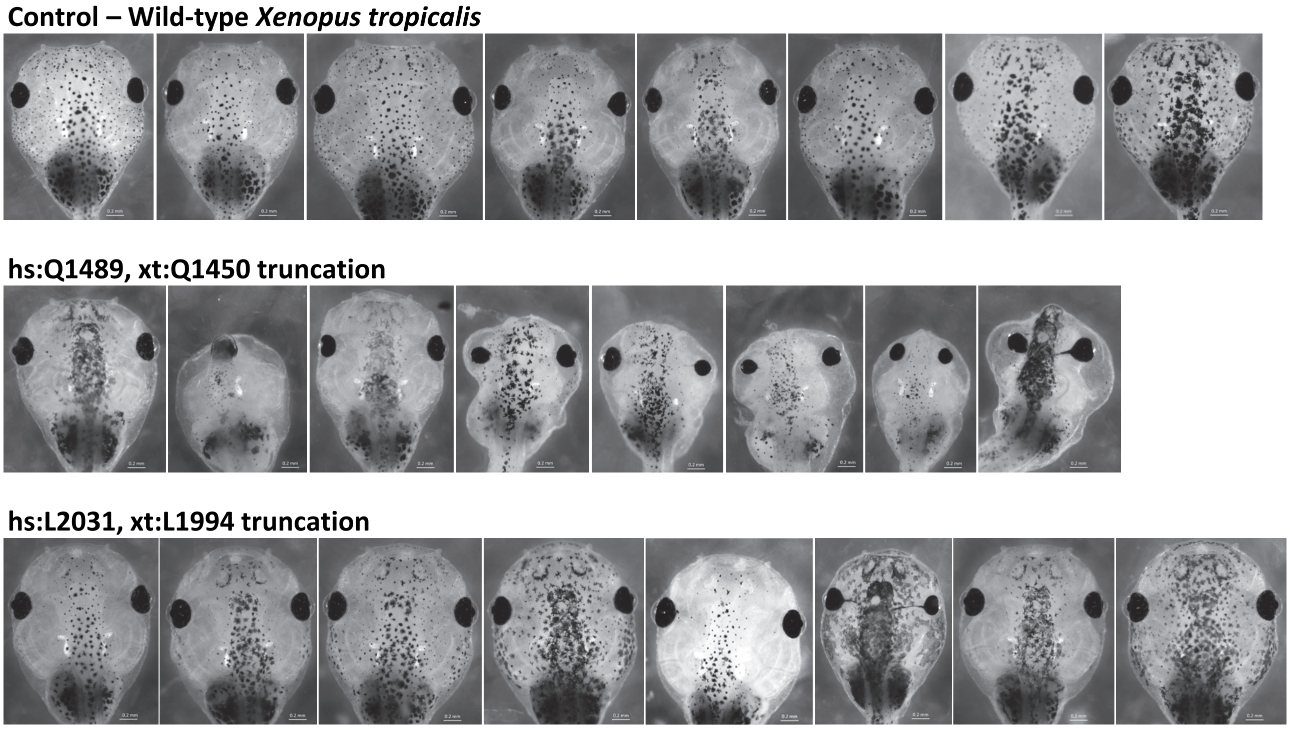

Figure S3: Truncation in the GEFD1 domain reduces head size in *X.tropicalis.*

A. Amplification of the target region followed by T7 endonuclease assay and Sanger sequencing confirmed that indels had been made at the target sites creating the truncations shown. **B.** Measurement of the head diameter of the injected tadpoles, 8 individuals per condition. **C.** Micrographs of all tadpoles used in the experiment.

Control – Wild-type *Xenopus tropicalis*

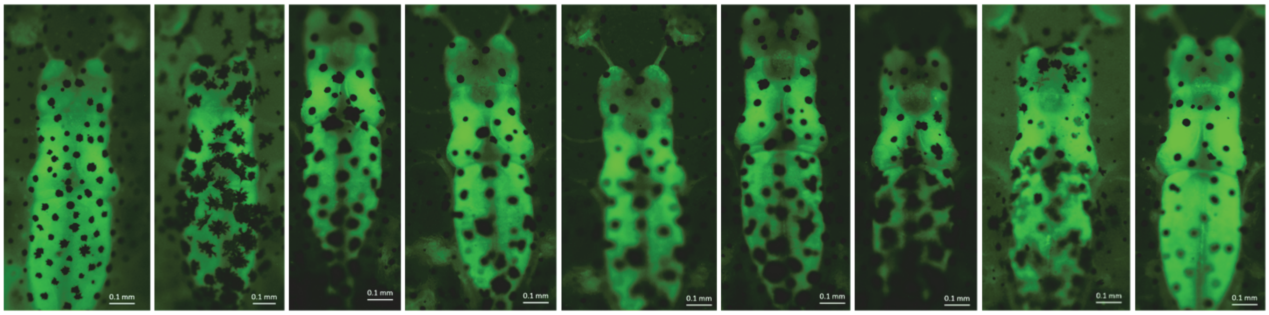

hs:Q1489, xt:Q1450 truncation

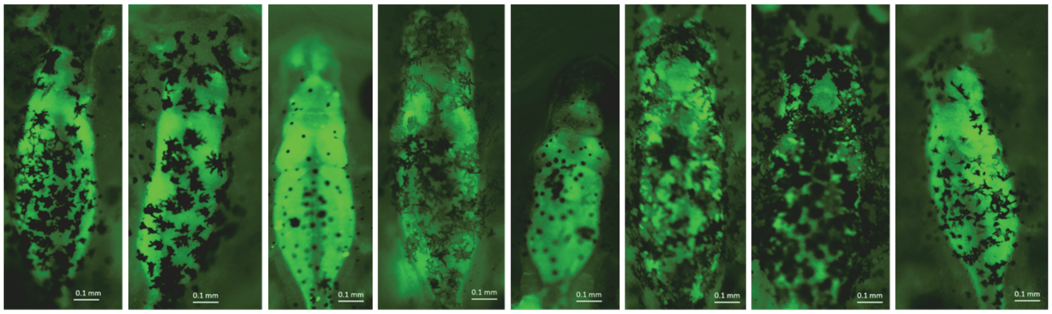

hs:L2031, xt:L1993 truncation

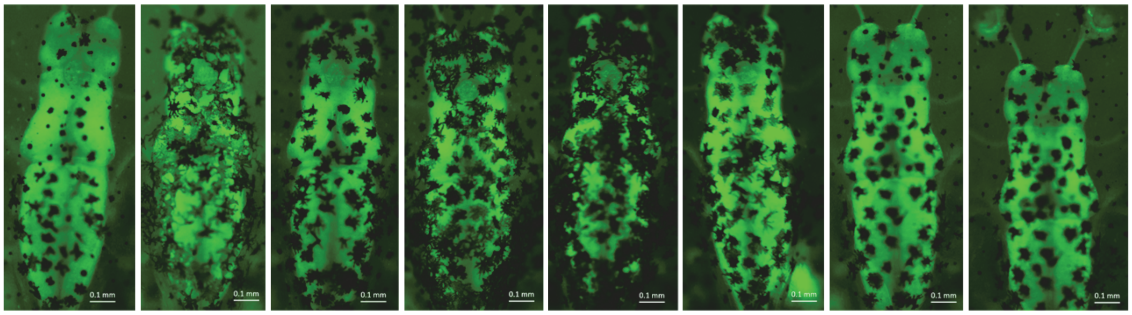

Figure S4: Truncation in the GEFD1 domain shows forebrain deformities in *X.tropicalis.*

GEFD1 and GEFD2 frameshifts were performed in a tubb2B.GFP transgenic *X. tropicalis* line, in order to examine gross structural abnormalities of the brain. This transgenic line labels differentiated neurons, and revealed forebrain deformities in the GEFD1 truncation.

Figure S4

trio single guide RNAs (Human, Xenopus tropicalis)	Genotyping PCR primers
Q1489*, Q1450	
taatacgactcactataGGTGAGAACATTGAATCTCAgttttagagctagaa	FWD: acaaacaactttaaaacccagaaaca
taatacgactcactataGGAAGAGTCTTTTCAAGTGTgttttagagctagaa	REV: ctgaaagcagctgagccagt
L2031*, L1993*	
taatacgactcactataGGTGAACTGGAAAGGTGCCgttttagagctagaa	FWD: atgctgcagaactgggattg
taatacgactcactataGGGTGAACTGGAAAGGTGCCgttttagagctagaa	REV: cctactcagcagggtcacag

Table S2: Oligonucleotides used to synthesise single guide RNAs and to amplify target regions of TRIO. Single guide RNA sequences were chosen using CRISPRscan; the target region itself is in uppercase and the oligonucleotides include a T7 RNA polymerase promoter in lowercase at the 5' end and part of the conserved region that binds Cas9 in lowercase at the 3' end. For the experiments shown here the upper sequence of each pair was used for both the GEFD1 and the GEFD2 truncating mutants.

	Age in Months						
	Domain	Min	1st Qu	Median	Mean	3rd Qu	Max	P value
First Words	Group 1	36	48	53	52	57	66	0.561
	Group 2	17	22.25	39	38.75	55.5	60	
Walking	Group 1	24	25.5	33	42	49.5	84	0.035
	Group 2	12	17	17	20.2	22	33	
Sitting	Group 1	7	9.75	11	17.36	24	36	0.168
	Group 2	8	9	9	9.33	9.75	11	
OFC	Group 1	53	54.3	56	55.98	57	59.5	<0.001
	Group 2	42	45	48	46.93	48.25	52	

Table S3: Statistical analysis of the developmental milestones of sitting, walking and speech, and ages achieved in either patient Group 1 of Group 2 mutation cases. Pearson's Chi-squared test was applied. Walking and OFC show a statistically significant difference between Group 1 and Group 2 individuals.
